# High efficiency adsorption of hexavalent chromium using bioderived activated carbon kinetics, isotherms, and thermodynamics

**DOI:** 10.1038/s41598-025-08472-7

**Published:** 2025-07-16

**Authors:** W. A. Hammad, Samah A. Hawash, Mohamed S. Abdel-latif, Mohammed Kuku, M. H. A. Amr

**Affiliations:** 1 Faculty of Engineering, Tanta University, Tanta, Egypt; 2 Department of Chemical Engineering, Menoufia Higher Institute of Engineering and Technology (MNF-HIET), Menoufia, Egypt; 3 Department of Mechanical Engineering, College of Engineering and Computer Science, Jazan University, Jazan, 45142 Saudi Arabia; 4 Engineering and Technology Research Center, Jazan University, P.O.Box 114, Jazan, 82817 Saudi Arabia; 5 Tanta Higher Institute of Engineering and Technology, Tanta, Egypt

**Keywords:** Adsorption, Hexavalent chromium, Palm fronds, Activated carbon, Wastewater treatment, Adsorption kinetics, Isotherm models, Surface characterizationL, Thermodynamic analysis, Environmental sciences, Chemistry, Energy science and technology, Engineering, Physics

## Abstract

Hexavalent chromium (Cr^6+^), a toxic pollutant in industrial wastewater, poses serious environmental and health risks. This study investigates H₃PO₄-treated palm frond-derived activated carbon (PFTACs) as a low-cost, sustainable adsorbent for Cr^6+^ removal. PFTACs achieved 99.64% removal efficiency within 90 min at 25 ± 1 °C, with strong performance across a pH range of 2–8. Surface analyses confirmed its mesoporous structure and high surface area, while FT-IR indicated physical adsorption as the dominant mechanism. The process followed pseudo-second-order kinetics and fitted the Langmuir isotherm, suggesting monolayer adsorption. Thermodynamic analysis showed an exothermic nature, with reduced adsorption at higher temperatures. These findings support PFTACs as an effective and environmentally friendly solution for Cr^6+^-contaminated water treatment.The novelty of this research lies in the development of H_3_PO_4_-treated palm frond-derived activated carbon (PFTACs) as an innovative, sustainable adsorbent for hexavalent chromium (Cr^6+^) removal. The use of palm fronds, an abundant agricultural waste, offers an eco-friendly and cost-effective alternative to traditional adsorbents, contributing to both waste reduction and efficient pollutant removal in industrial wastewater.

## Introduction

Water is vital to all living things, as well as to many other industries, farming, irrigation, personal cleanliness, and the industrial sector. When wastewater that has not been properly or even partially treated is released into the environment, it poses a risk to water resources. Prior to wastewater being released into the environment, it is crucial to remove any dangerous materials and maintain the water quality criteria established by applicable regulations^1^.

Most heavy metals are toxic to living, do not biodegrade, and tend to accumulate in biota. As a result, they impair immune function, induce cancer, and endanger life as we know it. This makes their presence in water resources an international concern^2^.

Due to their non-dissolving nature and essential characteristics that cause them to amass and concentrate on the numerous organs of the human and animal body, heavy metals are extremely dangerous substances. While some of these elements are necessary for proper metabolism, excessive concentrations of them in the human body are thought to be harmful. Industries are one of the main causes of heavy metal contamination in the environment. Furthermore, a lot of heavy metals build up in agricultural areas as a result of irrigating farms with untreated wastewater. This leads to a lot of health issues since the metals are transferred to human and animal bodies through food that is tainted by the heavy metals^3^ .

There are two types of causes for heavy metal pollution: artificial and natural. Natural causes include flooding, volcanic eruptions, and rock degradation. Human activities that release heavy metals into the environment include those from residential, commercial, mining, and agricultural sources. Human health is impacted by environmental heavy metal contamination.It is impossible to overstate the effects. Imagine human life being contaminated by heavy metals. Human exposure to heavy metals is linked to organ damage, including the skin, lungs, and heart. Humans with elevated manganese levels may develop Parkinson’s disease and movement difficulties. Additionally, anyone could exhibit hallucinations, cognitive impairment, and Amyotrophic Lateral Sclerosis (ALS)^4^.

Natural water contains two consistent types of chromium: Cr^+3^ and Cr^+6^. The oxidation state of chromium determines its solubility, stability, and toxicity. Compared to Cr (III), Cr (VI) is more soluble and poisonous, but Cr^+3^ can form complexes with organics to become bioavailable. Adsorption processes on iron oxide minerals determine the mobility, reactivity, and bioavailability of Cr^+6^ in soil settings. Cr (VI), or hexavalent chromium, is a frequent pollutant in groundwater. Human health will be greatly impacted by prolonged exposure to Cr (VI), which might result in cancer and deformity.The concentration of Cr^+6^ in natural wastewater is typically very low, ranging from 0.05 to 0.1 mg/L due to natural sources. However, in industrial wastewater, concentrations can be much higher, ranging from 1 to 50 mg/L, and may exceed 100 mg/L in heavily contaminated areas. Cr^+6^ is highly toxic, and even small concentrations can pose serious environmental and health risks. For drinking water, the EPA sets the maximum allowable level of Cr^+6^ at 0.1 µg/L^5^.

The textile, wood preservative, anti-corrosion, battery, paint, ink, chromate pigments, coatings that prevent corrosion and oxidation, leather tanning (using sodium dichromate), and ceramic coloring industries are among the sectors that employ hexavalent chromium^6^. Because of their ability to oxidize, all compounds containing hexavalent chromium are carcinogenic and poisonous^7^.

**Fig. 1:**
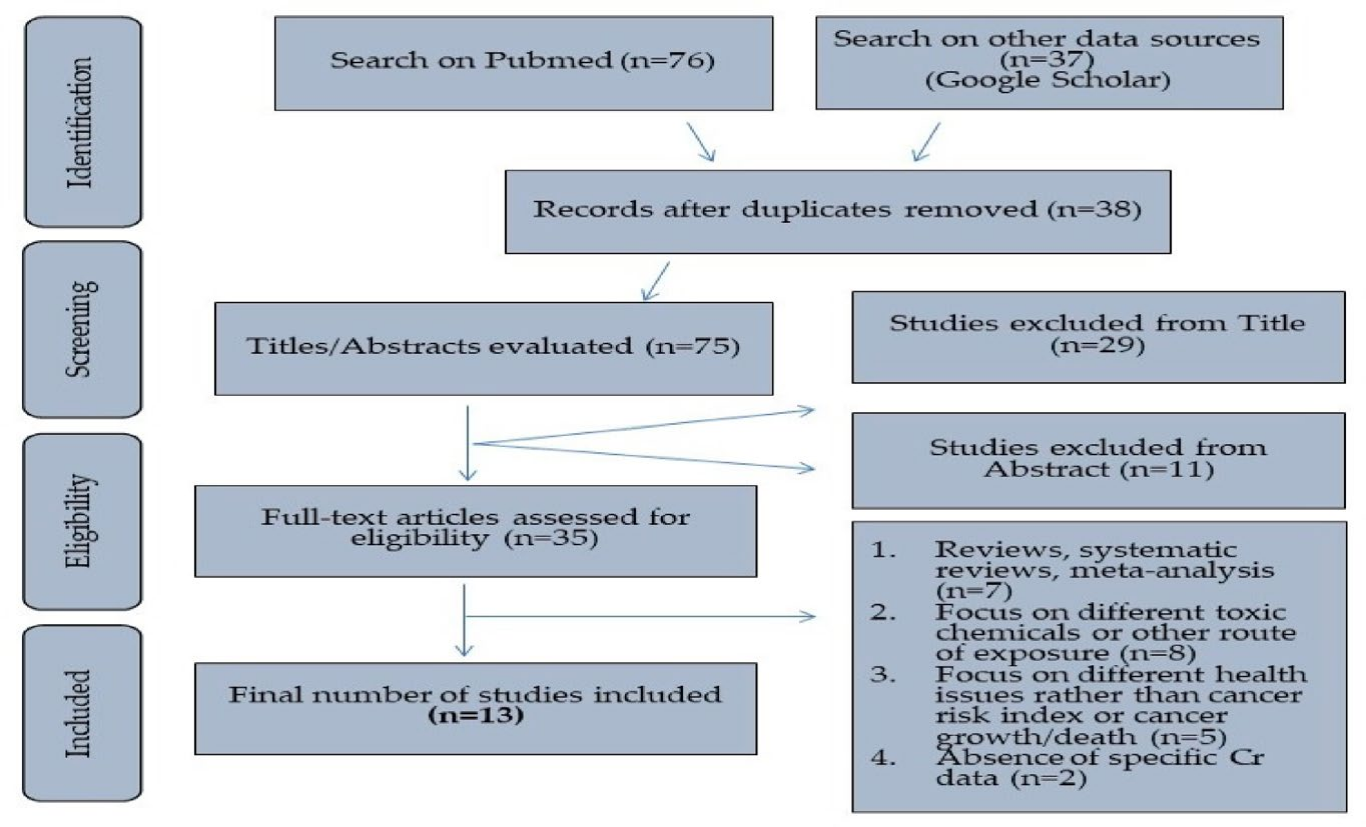
Flow diagram for finding and choosing relevant epidemiological research examining the connection between chromium exposure and cancer risk^(4)^^8^.

Figure 1 only looked at studies that focused on other exposure routes such as exposure from food and air or additional health consequences with a particular sample, such as skin conditions, rather than the association between water exposure to Cr^+6^ and the development of cancer. Comprehensive evaluations and meta-analyses of the risk of cancer associated with chromium in water and humans or studies of harmful substances^8^.

It is imperative removing these categories of water pollutants from wastewater prior to its release. In order to accomplish this, contaminated water has been remedied. Solvent extraction, ion-exchange procedures, chemical precipitation, oxidation or reduction reactions, membrane filtration, electrochemical treatment, adsorption, foam separation, and photocatalysis are some of the ways used to treat water^9^.

In terms of how well it removes heavy metals, adsorption is a very sustainable alternative therapy. The high adsorption capacity, common adsorbents such as activated carbon (AC), zeolite, and clay are widely used. According to studies, industrial metal-bearing effluents can be made more environmentally friendly by using agricultural wastes, which were previously thought to be a hazard to the environment. Agricultural wastes, including rice straw, pomelo peel, bean husk, raw pine cone, sawdust from durian trees, coconut coir, empty oil palm fruit bunches, and palm fronds^10^.

More than 60 fronds, each approximately 120 cm in length, are produced by each tree. Plant management practices, including trimming, fruit harvesting, and revitalizing the plant by removing the stems and fronds from the trees, produce palm oil fronds^11^.

Egypt has been growing date palms for thousands of years. In Egypt, the date palm tree is very beneficial both nutritionally and socioeconomically. It is a key fruit tree and the ideal crop to produce, such as exposure to food and air or additional health consequences with a particular sample, such as skin conditions, due to its ecological advantages in oasis agriculture and its historical use as a major source of food and byproducts. The world’s top prolific nation for date palm fruit is Egypt. There is a great chance to expand the date palm production area in order to meet domestic demand across the nation and generate date fruits for export^12^.

**Fig. 2:**
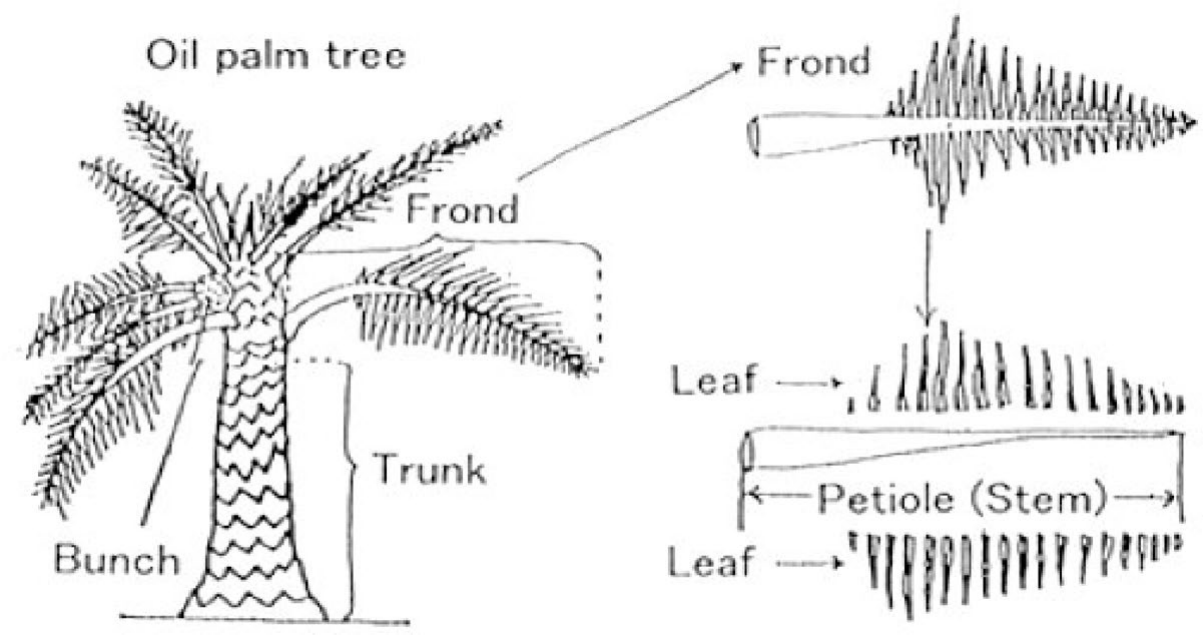
Anatomy of an oil palm frond (OPF)^8^.

Due to its abundance of palm trees, Egypt is the world’s top producer of dates, turning out a million seven hundred thousand tonnes of them annually. An estimated 15 million palm trees are shown in Fig. 2. One palm tree yields about 60 kg of dates per year, while the pruning of palm trees during the winter months results in an average of 15 kg of waste. There will be issues with the environment, economy, and health if the garbage is not disposed of safely. The following prospects for employment, health, and finances can be created from these wastes:

- Remaining palm leaves are utilized to make organic fertilizer.
- Production of livestock feed.
- The production of wood, furniture, and other materials for handicrafts, decorating, and basic home needs.
- “Biogas” or biogas produced from palm waste[^13,14^.

**Fig. 3:**
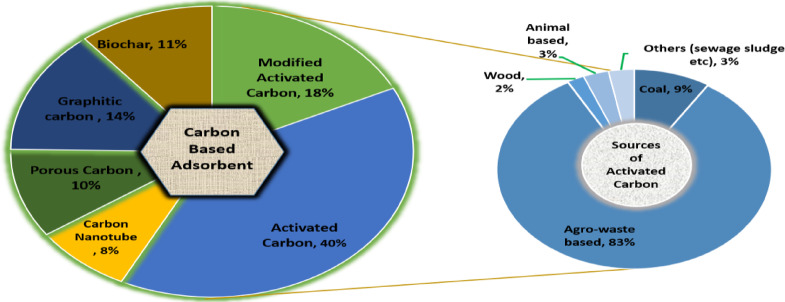
Sources of AC for water treatment^12^.

Although it is more expensive, Acs is typically accustomed to eliminating harmful contaminants from textile wastewater. As a result, efforts are being made to create inexpensive adsorbents from waste products from industry and agriculture. Recently, as demonstrated in Fig. 3, research has focused on employing a sorbent made of industrial waste to lower treatment costs while safeguarding the environment and public health^15^.

This study presents an innovative approach by utilizing H₃PO₄-treated palm fronds, an abundant agricultural waste, to develop a low-cost, eco-friendly activated carbon for the efficient removal of hexavalent chromium (Cr^6+^) from wastewater. Unlike conventional adsorbents, the synthesized material combines sustainability, high surface area, and mesoporosity, achieving an exceptionally high removal efficiency of 99.64% within a short contact time. The study also integrates comprehensive kinetic, isotherm, and thermodynamic analyses to elucidate the adsorption mechanism, demonstrating that the process is primarily physical, monolayer-based, and exothermic. This work advances the field by offering a green, scalable solution for heavy metal remediation, particularly suited for application in low-resource settings.

## Experimental study

### Materials and methods

The Research Lab of Fine Chemical Industries provided the chemicals used in this investigation. Throughout the experiment, solutions were prepared in accordance with established methods, and distilled water was used.

### Adsorbent material preparation

The palm frond samples were sliced into 1–3 cm sections. The parts were carefully cleaned to get rid of the dust, fibers, and other contaminants. Tap water and distilled water were used for the first wash. Samples of palm fronds were dried in a lenton oven at 105 °C for five hours in order to eliminate any moisture. The palm fronds (RDFs) that had dried were ground into a powder. To create (ACs), the powder was first impregnated with 60% H_3_PO_4_ solution. Five grams of samples Weighing the palm fronds, we combined them with 15 cc of 60% H_3_PO_4_. Then impregnated in the furnace for two hours at 100 °C, and they were subsequently activated in the stove for three hours at 400 °C. In order to remove the acidity from the manufactured ACs, the sample was washed. Until pH 7 was reached, the washing procedure was continued. After that, the samples were dried in an oven at 110 °C to eliminate any remaining moisture^12^.

### chromium stock solution preparation

To produce a stock solution with a chromium concentration of 1000 mg/l, 2.8 g of potassium dichromate was dissolved in 1000 ml of distilled water. To get different quantities of Cr^+6^ ions, starting at 1000 mg/l, the stock solution was diluted with distilled water in accordance with the dilution law. Every chemical utilized was 99.9% pure and of analytical quality. The investigations were conducted at a steady temperature of 25 °C, and 0.1N NaOH or 0.1N HCl were added to the solutions to bring their pH down to the necessary level.

### Adsorption experiment

**Fig. 4:**
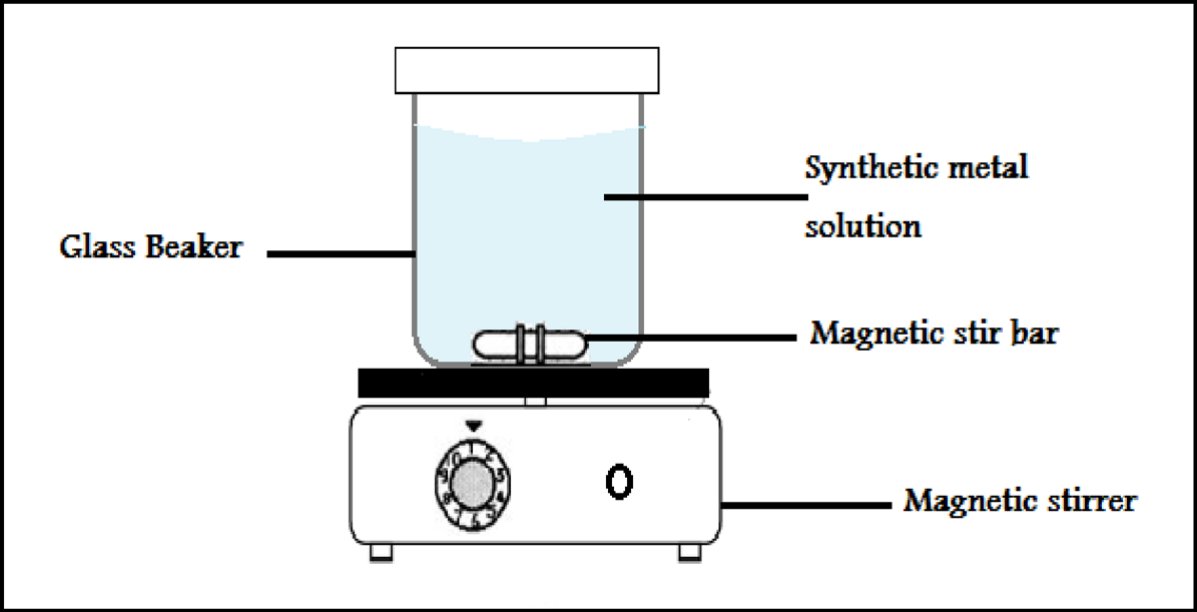
Schematic diagram of the apparatus used.

Using the batch equilibrium method, the adsorption experiments of Cr onto palm fronds treated with Acs with H_3_PO_4_ (PFTACs) were carried out in 250 ml of Cr^+6^. The following process parameters were applied to the adsorption experiments: Temperature: 20–45°C; initial metal concentration (C_0_): 50–300 mg/l; contact time(t): 5–90 min; pH 2–8; adsorbent dosage: 0.1–1 g. The pH of the initial solution was changed using a 0.1 M HCl or NaOH solution. The necessary mass of adsorbents was added to a beaker containing a Cr^+6^ solution. A ceramic hot plate stirrer (Model C230V50/60Hz) was then used to agitate the solution at 3000 rpm at the appropriate contact time to reach equilibrium. Figure 4 To extract adsorbents, a 5 ml solution was obtained at the end of the proper time interval and centrifuged (Model C230V50/60Hz) for 15 min at 4000 rpm. ICP-OES spectrometry was used to evaluate the metal concentrations in the supernatant.

The % adsorption has been calculated using Eq. (1)

1 $$\% Removal = \% adsorption = \left(\frac{{C}_{0}-{C}_{t}}{{C}_{0}}\right)\times 100\%$$

Co and Ct stand for the initial concentration of Cr^+6^ in ppm and the concentration of Cr^+6^ at equilibrium (after time t) in ppm, respectively^16^.

Using BELSORP max II equipment from Japan, the S.A. and porosity of the produced sample were calculated from N_2_ adsorption measured at − 176 °C. The sample was first out-gassed for 24 h (10–4 Torr) at 100 °C while under vacuum. The sample’s fine pore structure can be assessed once the pressure has grown to saturation, at which point all of the pores are filled with liquid. The condensed gas was subsequently evaporated from the system by gradually lowering the adsorptive gas pressure^17^.

## Results and discussion

### Characterization of solid adsorbate

**Fig. 5:**
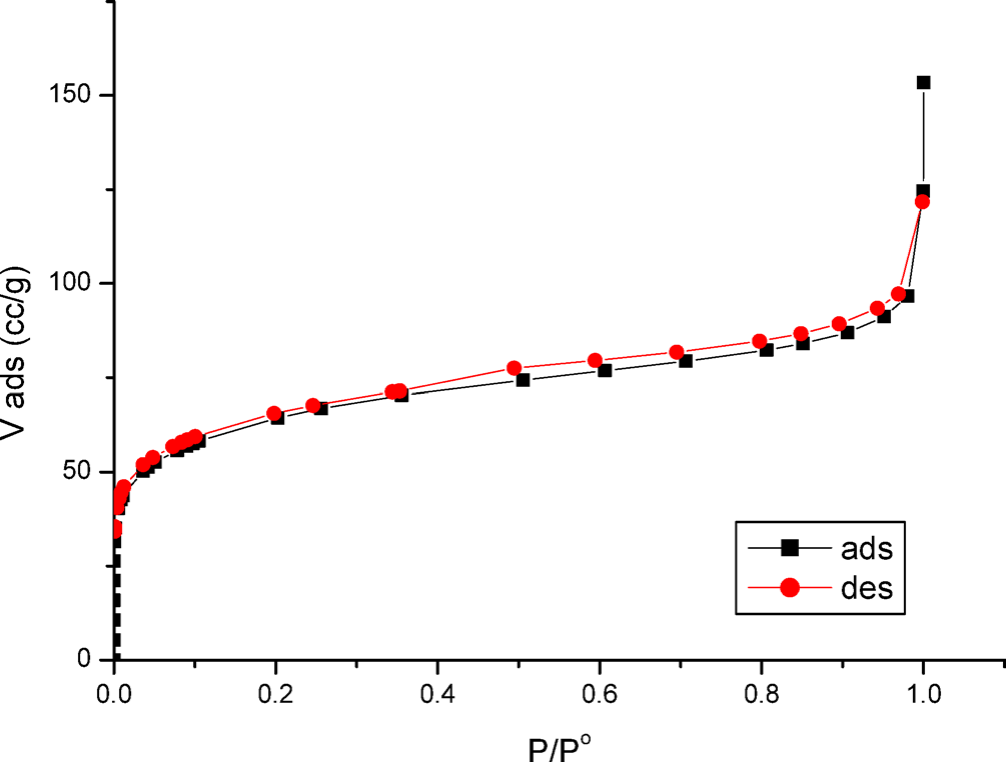
N_2_ adsorption–desorption isotherm before the adsorption processes.

Figure 5 shows the N_2_ adsorption–desorption isotherm plot for PFTACs prior to the adsorption procedures.

At high relative pressure, the Type IIb isotherm of PFTACs exhibits an H4 hysteresis loop. It is important to note that F. Rouquerol et al. proposed a new categorization, Type IIb, for isotherms that resemble Type IV but do not have a plateau at high P/P°^18^. The hysteresis loop that emerges within the P/P° range of 0.4 to 0.9 is frequently associated with capillary condensation in the mesopores^19^. Slit-shaped holes, primarily in the micropore range, are commonly observed in Acs, and their presence is confirmed by Types H4 hysteresis^20^.

**Fig. 6:**
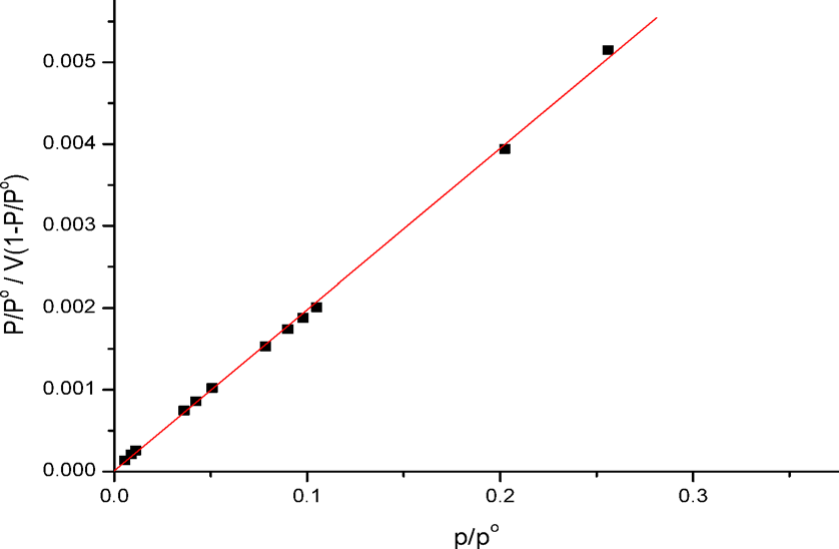
BET S. A plot before adsorption.

The best correlation between the equilibrium curves should be found in order to maximize the adsorption system. Figure 6 shows the linearised model of N_2_ multilayer adsorption relative to pressure using the BET method.

To calculate the overall specific S.A in m^2^/g it includes assessments of the exterior area and the pore area. This is how the BET equation is typically expressed:

2 $$\frac{P/{P}^{o}}{V[1-P/{P}^{o}]}=\frac{c-1}{{V}_{m}} \left(\frac{P}{{P}^{o}}\right)+ \frac{1}{{V}_{m }C}$$

where V is the amount of adsorbed gas, Vm is the amount of monolayer adsorbed gas, C is the BET constant, and P and Pm are the equilibrium and saturation pressures of the adsorbate at the adsorption temperature, respectively.

Based on experimental findings : SBET = 352.66 m^2^/g; VT = 91.415 cc/g; r = 8.02 nm; Vm = 80.7 cc/g; C = 1.002. It is noteworthy that a date stone-based ACs with a larger S.A was produced by activating it with H_3_PO_4_ acid^21^.

Pore Size Distributions (PSDs) over the mesopore and a portion of the micropore range were calculated using the pore size model created by Barret, Joyner, and Halenda (BJH). BJH pore size, where rp denotes the pore’s real radius, is equal to r_K_ + t. t is the adsorbed film’s thickness, and r_K_ is the pore’s Kelvin radius.

**Fig. 7:**
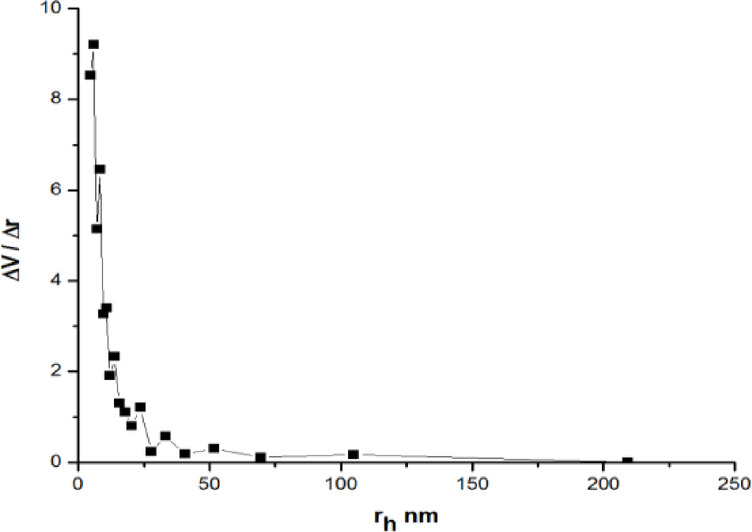
Pore size distribution by BJH before adsorption.

The decrease in the statistical thickness of the pores throughout the desorption process is taken into consideration by BJH. Figure 7 displays the sample’s pore size distributions as determined by the BJH technique on the desorption branches of isotherms. It shows the distribution of pore sizes in the mesopore area.

**Fig. 8:**
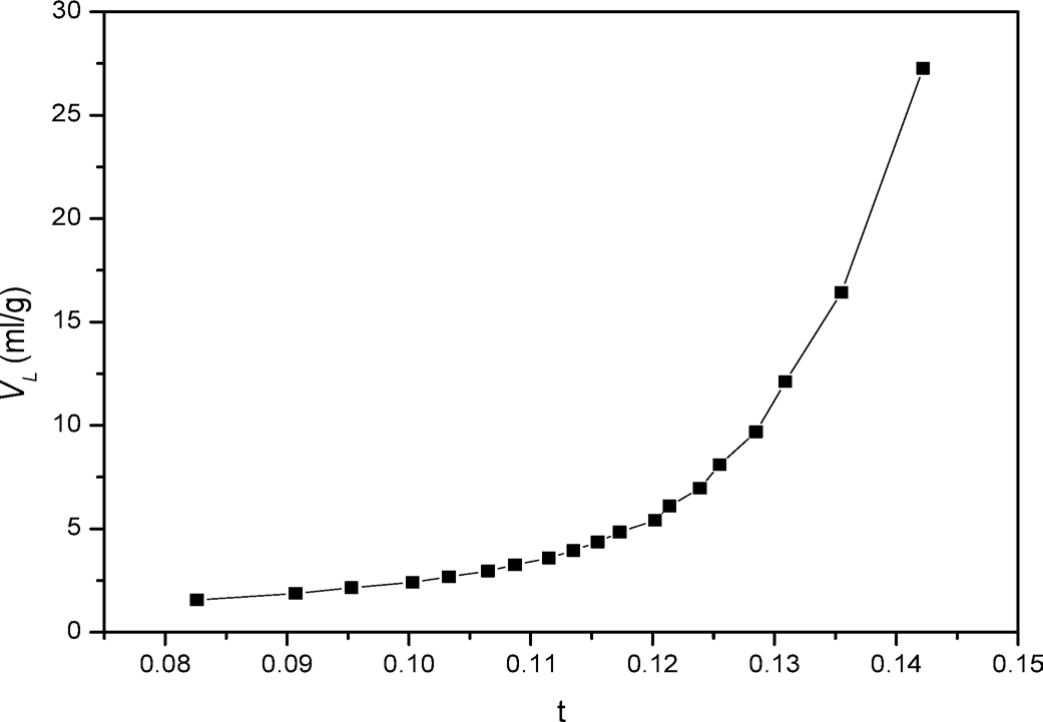
t-Plots before adsorption.

To determine the exterior surface area, micropore volume, and total pore volume, the experimental data was analyzed using the t-plot method. Layer thickness “t” as a function of rising p/po was calculated using a mathematical model of the multi-layer. Figure 8 As pressure increases, so does the adsorbate layer’s thickness, or “t.” Fig. 4 plots the experimental volume adsorbed (V_L_) against statistical thickness (t) for every p/p^o^. According to Lippens and de Boer’s classification, the sample shows upward deviation, indicating the existence of meso-porosity. The S.A S_t_(m^2^/g) was calculated from the slope of the straight lines passing through the origin; these values are identical to the S_BET_ value^22^.

**Fig. 9:**
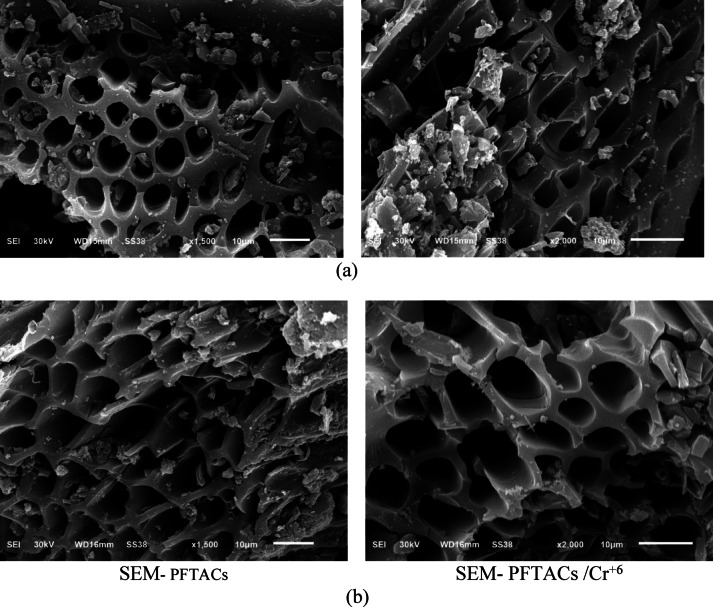
(**a**) SEM PFTACs and (**b**) (SEM) of Cr^+6^ adsorbed on PFTACs (PFTACs /Cr^+6^).

The morphology studied by SEM (Fig. 9) shows that the samples of PFTACs and Cr^+6^ adsorbed on PFTACs (PFTACs /Cr^+6^) were porous. The topography of the surface matches the value of S.A. and porosity from BET, t-plot, and BJH measurements. The image reveals the material’s big, dispersed pores. It is possible that the material underwent chemical and thermal treatment to increase its capacity for absorption or reactions, which led to the formation of these pores. It is determined that the material, which appears to be a form of activated carbon based on what is seen in the image, has a high porosity, which indicates that it has a large S.A. and is, therefore, useful in processes like adsorption and absorption.

Following the adsorption process, image (B) may show some minor surface modifications, such as tiny deposits or extra materials adhered to the pore surface. These deposits could be the consequence of chromium buildup on the substance. The pore structure might be impacted by chromium adsorption. A substantial amount of adsorption could cause some pores to shrink or partially close, as seen in the second image, which has more homogenous pores than the first. This shows the effect of chromium on the substance and the effectiveness of adsorption. These photos provide tangible proof of the alterations the adsorption process caused to the material’s surface.

**Fig. 10:**
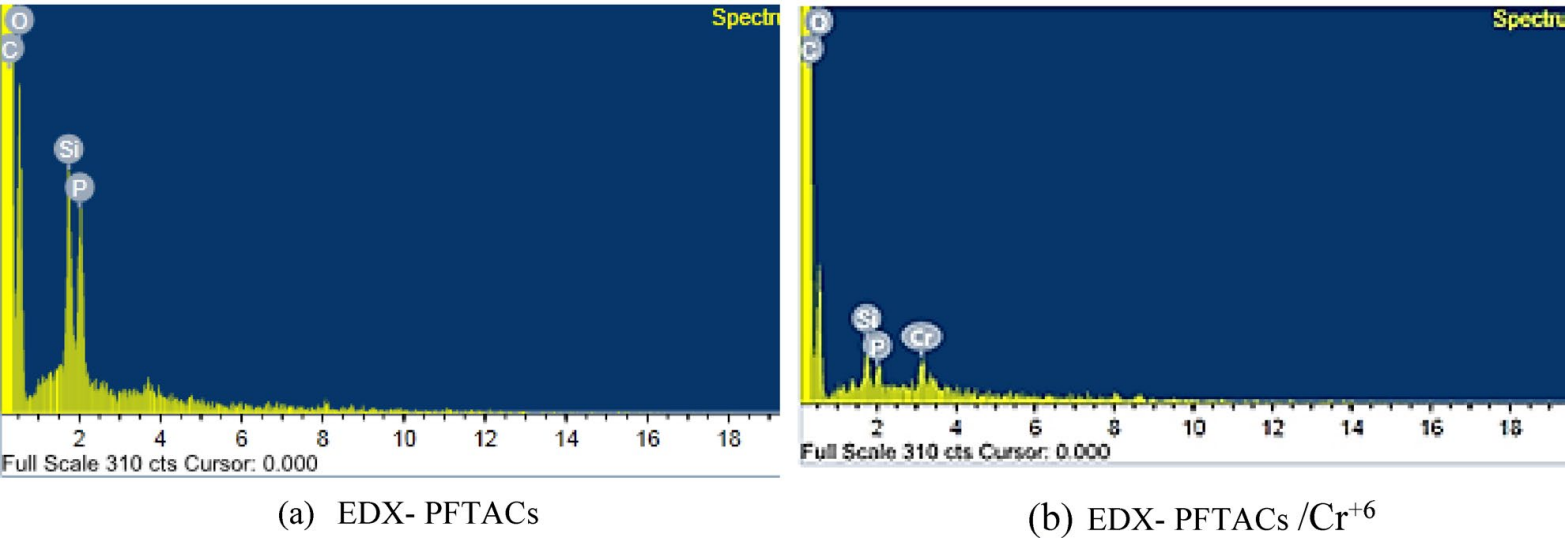
(**a**) EDX of PFTACs, and (**b**) Cr^+6^ adsorbed on PFTACs.

The SEM–EDX analysis (Fig. 10) of the sample adsorbed Cr^+6^ confirmed the presence of Cr^+6^ with a concentration of 2 wt%. Analysis of Major Peaks: The carbon peak appears at an energy level of around 0.3 keV.The oxygen peak appears at an energy level around 0.5 keV. The presence of oxygen may indicate metal oxides or materials containing hydroxyl (OH) groups.:The silicon peak appears at an energy level around 1.7 keV.The phosphorus peak appears at an energy level of around 2 keV.Carbon and oxygen have relatively high peaks, indicating a significant presence of these elements in the sample. Silicon and phosphorus have lower peaks, suggesting lower concentrations.

Material Composition: Based on the peaks, the material appears to have a high content of carbon and oxygen because it is an organic material. Silicon and Phosphorus presence indicates that the material contains silicate or phosphate compounds. Because our material is ACTPFs, the presence of carbon suggests that the material is indeed rich in carbon, as expected. Silicon and phosphorus could be related to some processing steps or residual materials from reactions. The chemical treatment of Palm Fronds with H_3_PO_4_. The appearance of chromium (Cr^+6^) in the second EDS spectrum is likely responsible for the observed reduction in the relative concentrations of silicon (Si) and phosphorus (P). Cr^+6^peak, observed at approximately 5.4 keV in the spectrum, explains the high absorption of Cr^+6^ on the surface of ACTPFs^23,24^.

**Fig. 11:**
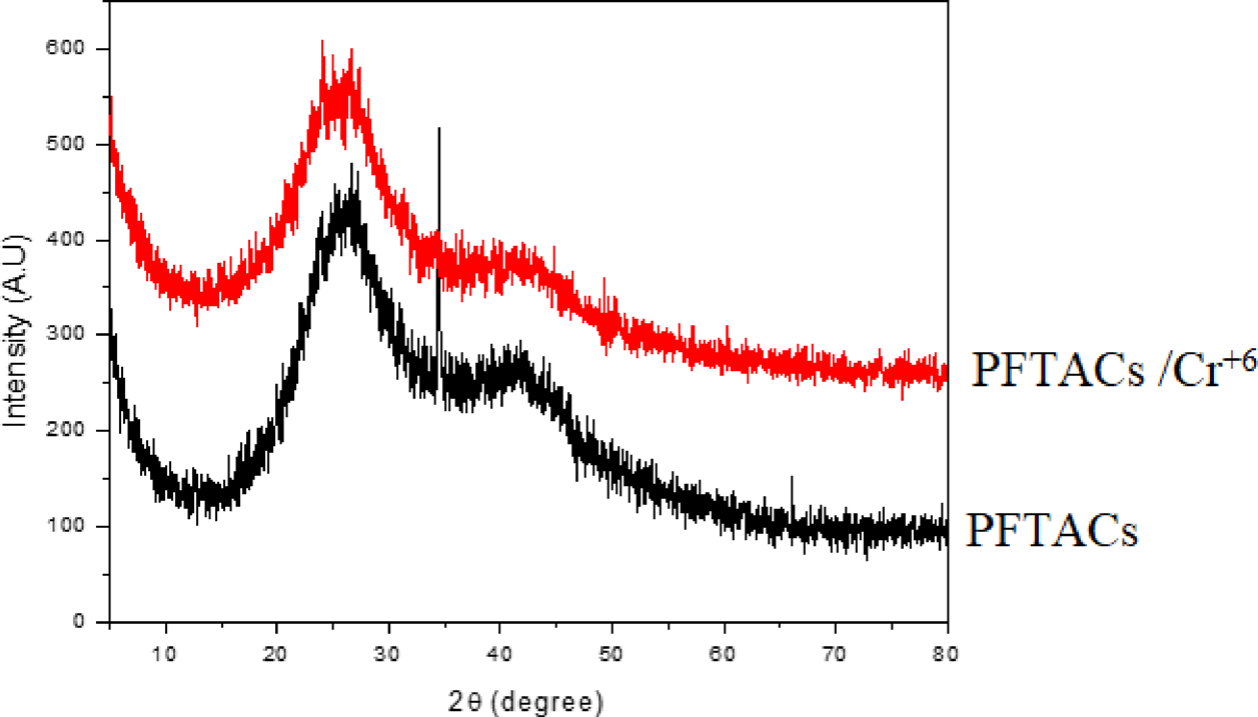
XRD of PFTACs and Cr^+6^ adsorbed on PFTACs (PFTACs/Cr^+6^).

The XRD pattern for PFTACs and Cr + 6 adsorbed on PFTACs (PFTACs /Cr^+6^) samples using CuKα (λ = 1.54 Å) as a radiation source with a 2θ scanning range from 5 to 80 is displayed in Fig. 11. The large peak in the diffraction pattern at 2θ = 26 suggests that PFTACs are amorphous and that Cr^+6^ is adsorbed on PFTACs (PFTACs /Cr^+6^). Furthermore, there is no organised crystalline structure visible in the recorded XRD pattern^25^.

**Fig. 12:**
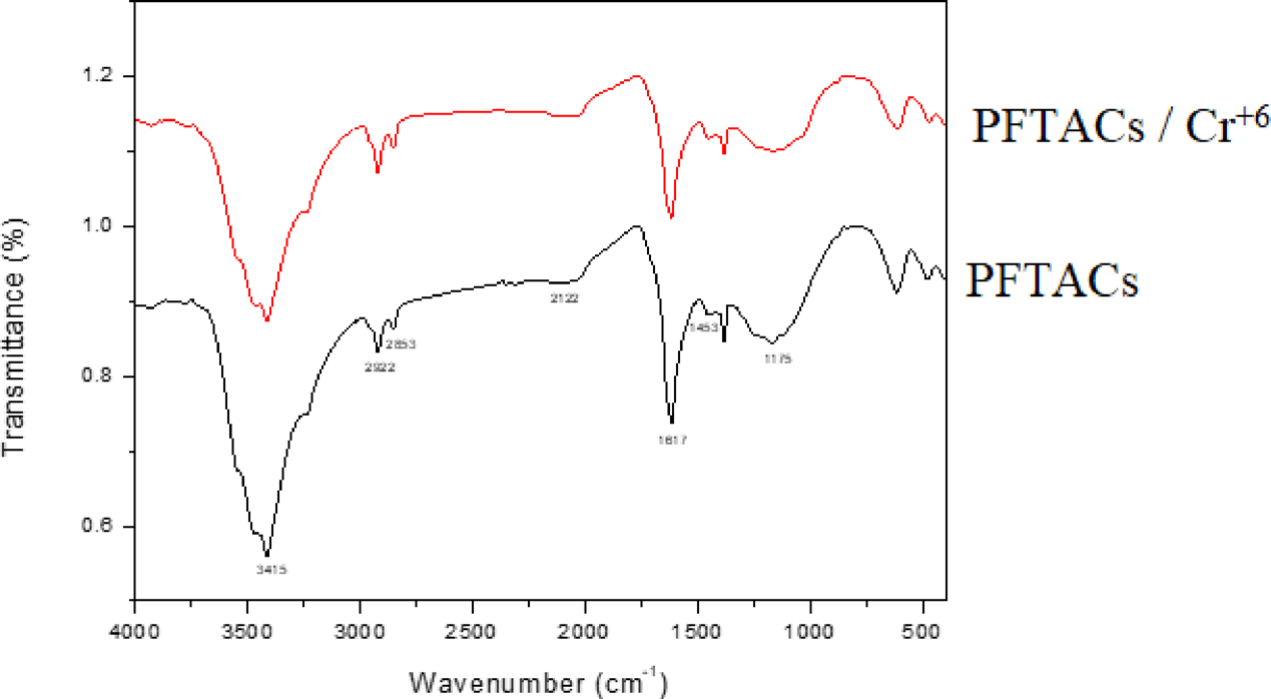
FT‒IR of PFTACs and Cr^+6^adsorbed on PFTACs (PFTACs /Cr^+6^).

Figure 12 displays the FT-IR (KBr, cm^−1^) of PFTACs after burning at the following wavelengths: 3415 cm^−1^ (OH), 2922 and 2853 cm^−1^ (CH-from.), 2122 cm^−1^ (CH-aliphatic.), 1617 cm^−1^ (C=O stretching of acid), 1453 cm^−1^ (C=C stretch), 1175 cm^−1^ (C–H bending vibration). FTIR indicates the presence of flavonoids and polyphenols in PFTACs. The FT-IR of PFTACs and Cr^+6^ adsorbed on PFTACs (PFTACs /Cr^+6^) did not change. That means the adsorption is physical.

The study provides a detailed explanation of the underlying mechanism of Cr^+6^ adsorption on PFTACs (H₃PO₄-treated palm fronds-derived activated carbon), focusing on various structural and chemical aspects that contribute to the adsorption process. At high relative pressure, the Type IIb isotherm of PFTACs exhibits an H4 hysteresis loop, which is indicative of capillary condensation in the mesopores. This loop emerges within the P/P° range of 0.4 to 0.9, a characteristic often observed in materials with mesoporous structures. Pore size distributions calculated using the BJH method highlight the presence of pores primarily in the mesopore range, further supporting the material’s potential for efficient adsorption.

The BET surface area of PFTACs was measured at 352.66 m^2^/g, and this high surface area aligns with its notable adsorption capacity for Cr^+6^. The t-plot analysis, in combination with BET, was used to determine the external surface area, micropore volume, and total pore volume, confirming that the material is highly porous and thus suitable for adsorption applications. These findings demonstrate that the surface structure of PFTACs plays a crucial role in enhancing the material’s adsorption efficiency.

In addition to surface area considerations, the SEM images of PFTACs before and after the adsorption of Cr^+6^ reveal significant changes in the material’s morphology. The porous nature of the material is evident, and minor surface modifications, such as small deposits adhering to the pore surface, are visible after the adsorption process. These modifications indicate the accumulation of Cr^+6^ on the surface, with the possibility that the adsorption process may lead to shrinkage or partial closure of some pores due to chromium adsorption. This change in the pore structure is an important indicator of the material’s effectiveness in removing Cr^+6^ from solution.

The SEM–EDX analysis further confirmed the adsorption of Cr^+6^ by showing its presence on the surface of PFTACs. The reduction in the relative concentrations of silicon and phosphorus post-adsorption suggests that chemical interactions, possibly involving functional groups on the surface of the material, may play a role in the adsorption process. FT-IR analysis also supports this conclusion by showing that the adsorption of Cr^+6^ is primarily physical, with no significant changes in the functional groups of the material, such as OH, CH, and C=O bonds, after adsorption.

The XRD pattern of PFTACs and Cr^+6^ adsorbed on PFTACs shows that the material remains amorphous, with no evidence of crystalline structures before or after adsorption. This lack of crystalline peaks suggests that the adsorption process does not induce any significant changes in the material’s crystalline structure, a common feature in activated carbon materials.

In conclusion, the adsorption of Cr^+6^ onto PFTACs is primarily driven by physical interactions, with the material’s high surface area, mesoporous structure, and chemical composition playing key roles in the adsorption process. The observed changes in pore structure and surface morphology after adsorption further underscore the material’s effectiveness in adsorbing Cr^+6^ from aqueous solutions, providing valuable insights into the adsorption mechanism.

### The effect of pH

**Fig. 13:**
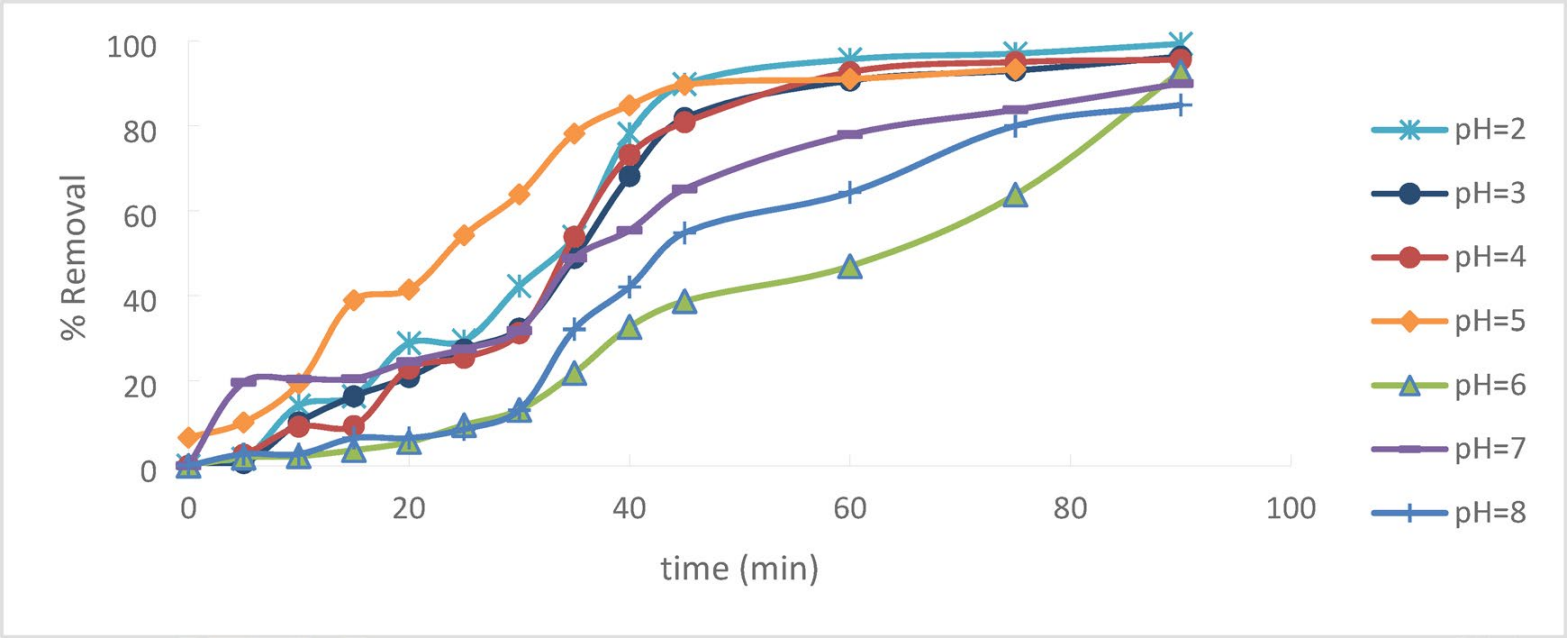
pH effect regarding Cr^+6^ adsorption onto (PFTACs) (C_o_ = 50 ppm, agitation speed = 300 rpm ,(PFTACs) dose = 0.5 g/250 ml, T = 25 °C and t = 90 min).

The solution’s pH is a critical factor influencing the extraction of chromium Cr + 6 from an aqueous solution and is responsible for the impacts on the adsorbent surface charges, ionization degree, and Adsorbate specification. The initial Cr^+6^ concentration and the contact time regarding adsorption are said to be 50 ppm and 90 min, respectively, to achieve complete equilibrium. The equilibrium studies at varying pH values of 2 to 8 are employed to understand the pH impact on Cr^+6^ adsorption. HANNA pH 211-Romania pH-meter was used for adjusting the pH of solutions.Fig. 13

**Fig. 14:**
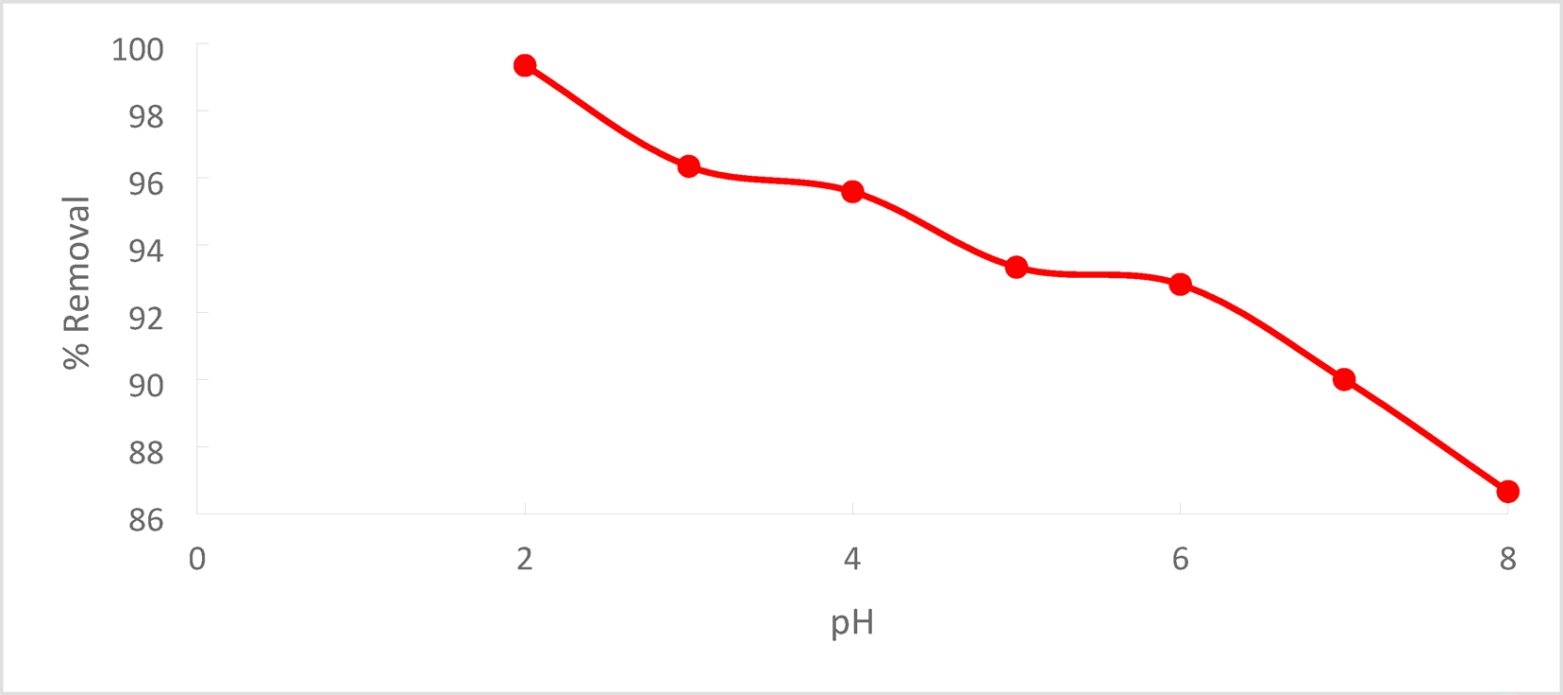
pH effect regarding Cr^+6^ adsorption

Increasing in pH will decrease the % removal,^26^ Fig. 14. Because of the low concentration of hydroxyl groups in the solution and the higher positive charge in Acs, the adsorption capacity was high at lower pH values. The electrostatic force for removing chromate ions from the solution, such as chromate (CrO_4_)^-2^, dichromate (Cr_2_O_7_)^-2^, and acid chromate (HCrO_4_)-1, is increased by such positive charge increases^27^. demonstrates that the amount of adsorbed Cr ions reduces with increasing pH, with maximal adsorption taking place at pH = 2 when the percentage removal of Cr^+6^ drops dramatically from 99.35% to 86.67%. For pH values between 2 and 8^28^.

### Effect of initial concentration

**Fig. 15:**
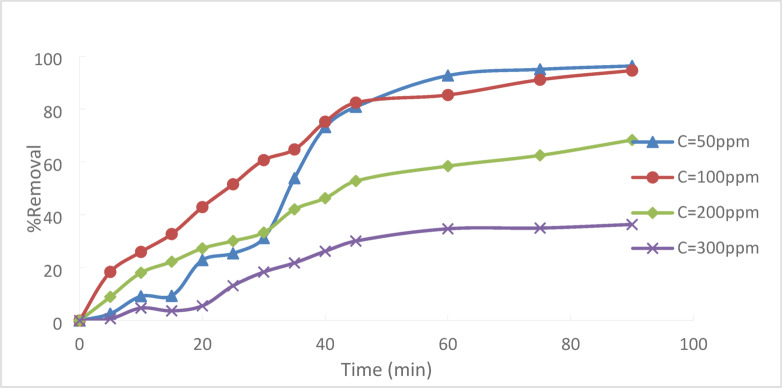
Effect Cr^+6^ C_o_ onto (PFTACs) (pH = 2,agitation speed = 300 rpm, (PFTACs) dose = 0.5 g/250 ml, T = 25 °C and t = 90 min).

Under controlled circumstances, the impact of C_0_ of Cr^+6^ on its removal from (PFTACs) was investigated. T = 25 °C, a pH of 2, a contact time of 90 min, and an agitation speed of 300 rpm were all maintained. For various Cr + 6 initial concentrations (50, 100, 200, and 300 ppm), the adsorbent dose is equal to 0.5 g/250 ml. Figure 15 illustrates that the percentage removal of Cr^+6^ falls as the initial Cr^+6^ concentration rises. Cr^+6^ is absorbed at low concentrations on the adsorbent surface’s empty sites, which are then saturated and filled as the concentration increases. Many active sites on the adsorbent’s surface become available for heavy metal adsorption when C_o_ of Cr^+6^ is low. The number of moles of Cr^+6^ is greater than the number of empty sites, though, if the starting concentration of Cr^+6^ is raised. As a result, the dye clearance rate drops, and the accessible sites are rapidly saturated^1–29^.

**Fig. 16:**
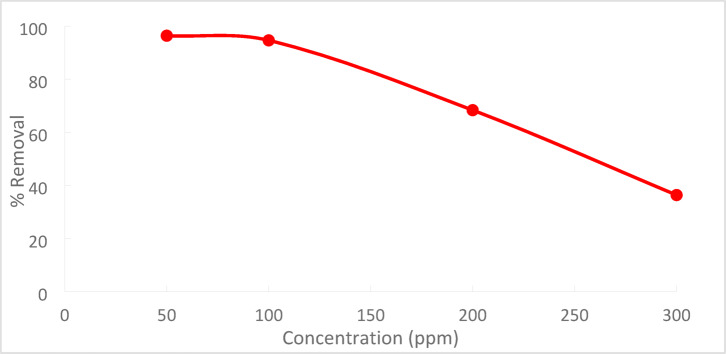
Effect of Cr^+6^ initial concentration on adsorption

As illustrated in Fig. 16, the percentage clearance of Cr^+6^ dropped from 96.3 to 36.36% when the initial concentration of Cr^+6^ rose from 50 to 300 ppm.

### The effect of the adsorbent dose

**Fig. 17:**
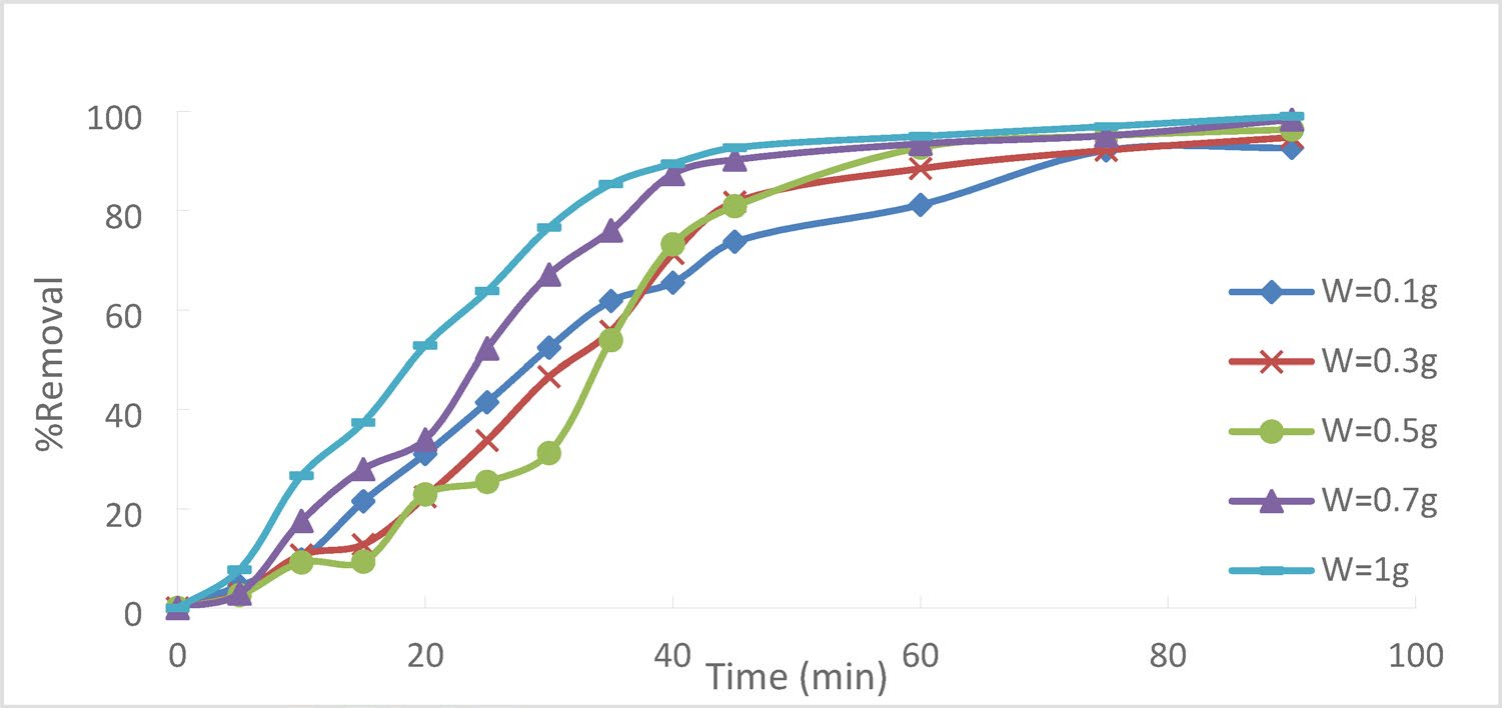
Effect of PFTACs dosage on the % removal Cr^+6^ (pH = 2,agitation speed = 300 rpm , (PFTACs) dose = 0.5 g/250 ml, T = 25 °C and t = 90 min).

At a starting Cr^+6^ concentration of 50 ppm, 90 min of contact, and T = 25 °C, the effect of the adsorbent dosage on the removal of Cr^+6^ was assessed. PFTACs = 0.1, 0.3, 0.5, 0.7, and 1 g/250 ml are the various adsorbent dosages. Figure 17 illustrates that as the amount of adsorbent used increases, so does the percentage removal of Cr^+6^. This outcome is anticipated as a greater S.A with accessible adsorptive sites is the result of increasing the amount of adsorbent, which means that increased availability results in more exchangeable sites or S.A guaranteed, which improved Cr^+6^ uptake^30,31^.

**Fig. 18:**
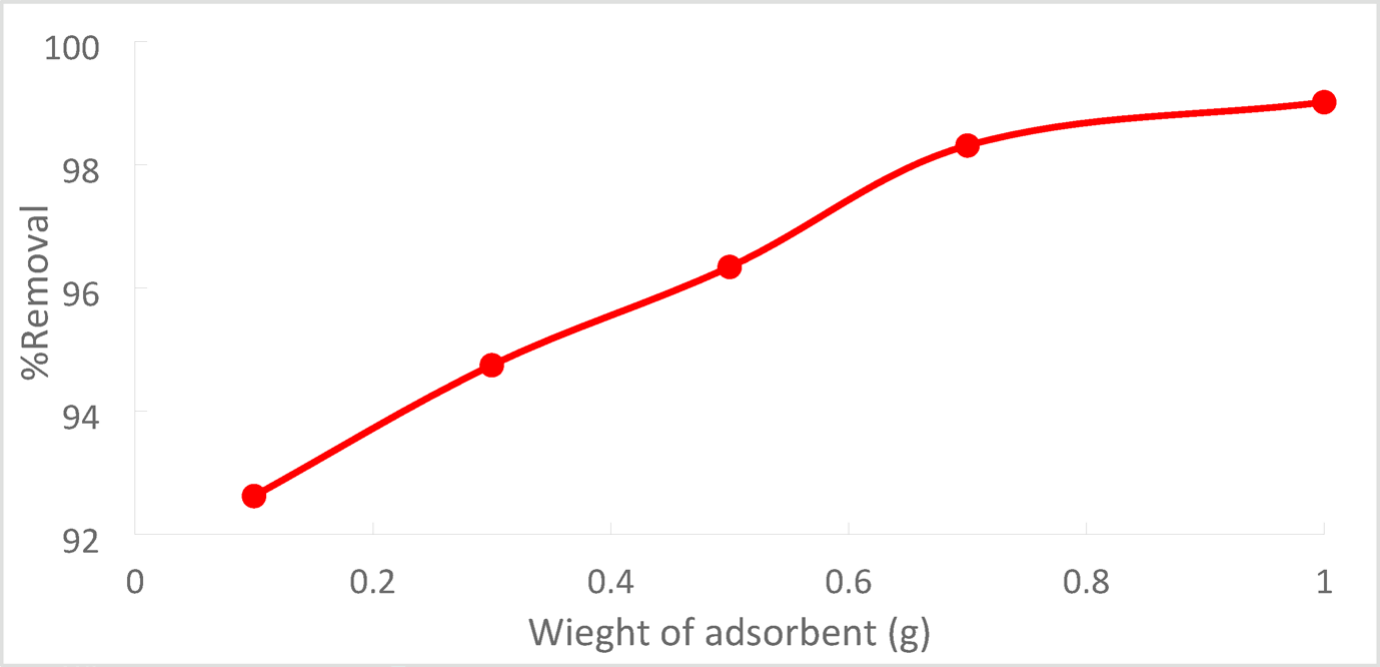
Effect of PFTACs on % removal Cr^+6^

As PFTACs rose from 0.1 g/250 ml to 1 g/250 ml, the absorption or percentage removal of Cr^+6^ increased from 92.63 to 99.01%, as shown in Fig. 18.

### The effect of temperature

**Fig. 19:**
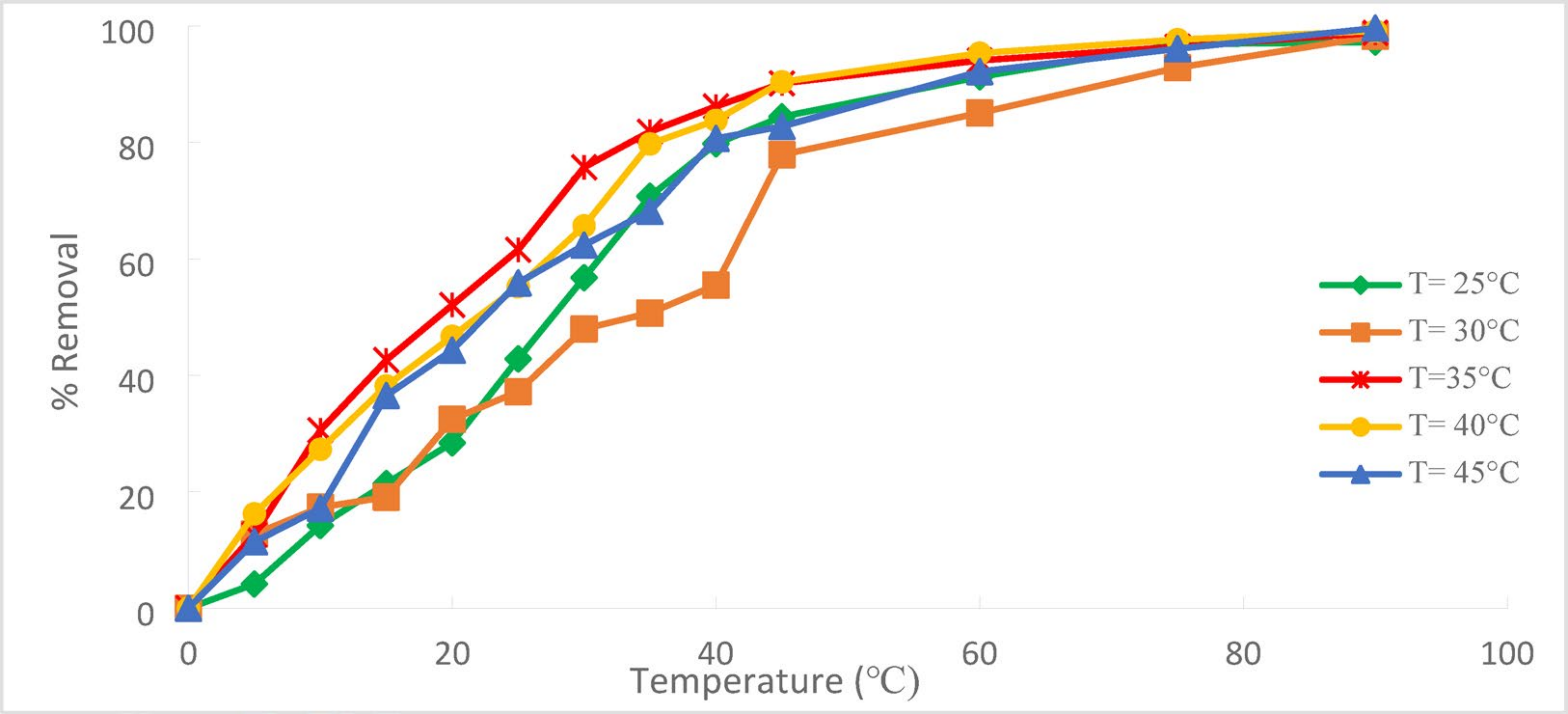
Temperature effect on % removal of Cr^+6^ on (PFTACs), Cr^+6^ C_o_ = 50 ppm, agitation speed = 300 rpm , (PFTACs) dose: 1 g\250 ml , t = 90 min and pH = 2.

The temperature effect on the percentage of Cr^+6^ removed from the solution using PFTACs is shown in Fig. 19. This investigation used a range of temperatures (25, 30, 35, 40, and 45 °C) and C_o_ 50 ppm of Cr^+6^ in the presence of (1 g/250 ml) for (PFTACs). According to the figure, the removal efficiency rose with temperature within 90 min.^32^ because higher temperatures promoted adsorption at the Cr^+6^ adsorbent’s coordination sites, which made it easier to remove Cr^+6^. This resulted from the formation of more activation sites on the adsorbent surface and the acceleration of some previously slow stages^33^.

**Fig. 20:**
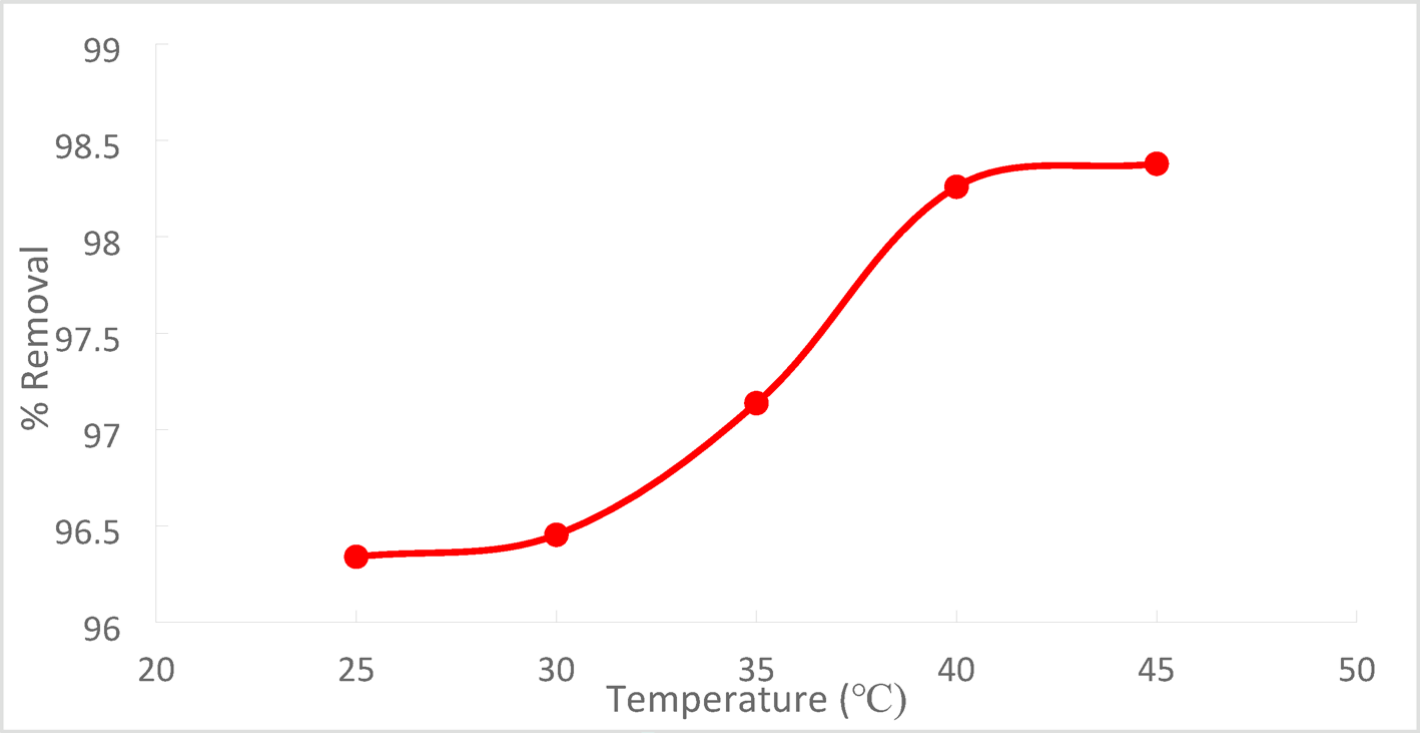
temperature effect on % the removal of the Cr^+6^

As the temperature was raised from 25 to 45 °C, the percentage removal of Cr^+6^ increased from 97.22 to 99.64%, according to the results shown in Fig. 20.

### Adsorption of kinetics

#### Pseudo-first order model (PFO)

The PFO equation describes the adsorption rate. Equation (4) is PFO kinetic model that (Lagergren, 1898) proposed:

3 $$\text{Ln }\left({\text{q}}_{\text{e}}-{\text{q}}_{\text{t}}\right)=\text{Ln}\left({\text{q}}_{\text{e}}\right)-{\text{k}}_{1}\text{t}$$

the form in Eq. (3) with boundary conditions of t = 0, q_t_ = 0 and t = t

4 $${\text{q}}_{\text{t}}=\frac{({\text{C}}_{0}-{\text{C}}_{\text{t}})\times \text{v}}{\text{m}}$$

5 $${\text{q}}_{\text{e}}=\frac{({\text{C}}_{0}-{\text{C}}_{\text{e}})\times \text{v}}{\text{m}}$$

**Fig. 21:**
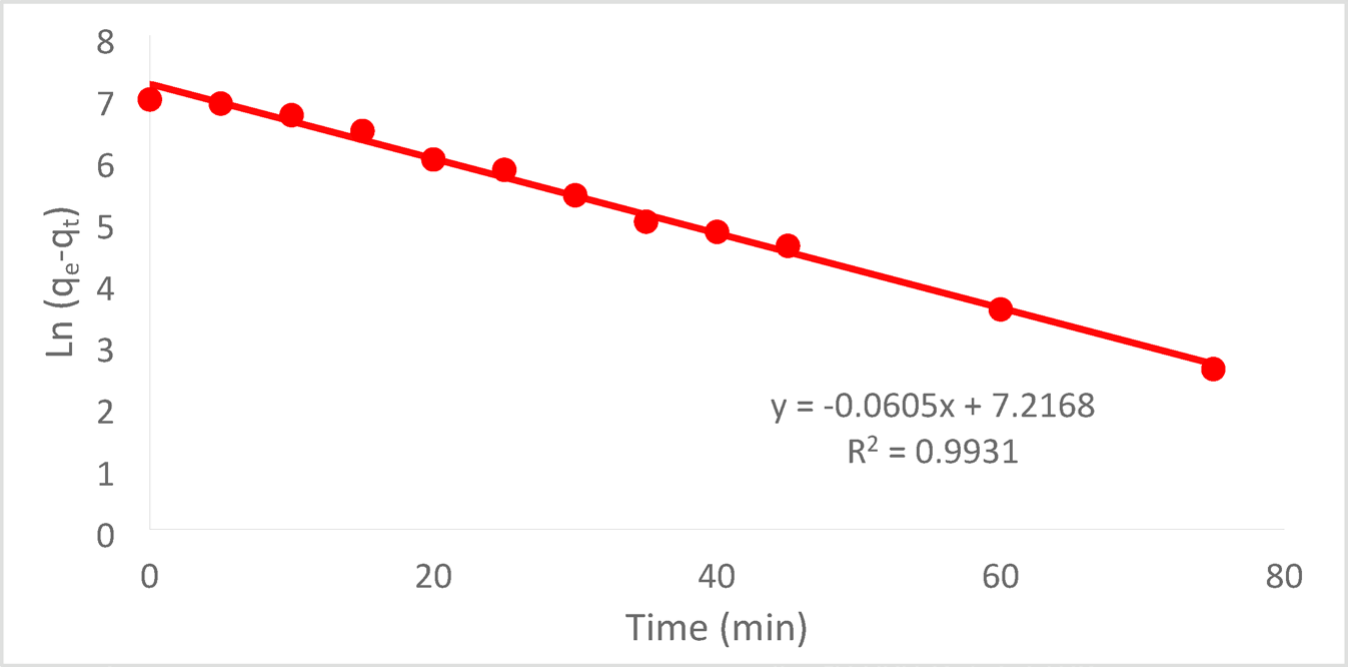
PFO kinetic fit for Cr^+6^ plots for the adsorption onto (PFTACs) at (C_o_ = 50 ppm, agitation speed = 300 rpm ,(PFTACs) dose = 0.5 g/250 ml,T = 301 K and t = 90 min, pH = 2).

Figure 21 illustrates a linear relationship between time (t) and the natural logarithm of (q_e_ − q_t_). The graph’s intercept and slope can be used to compute the parameters qe and k_1_, respectively^30^.

#### Pseudo-second order model (PSO)

The PSO kinetic rate can be determined using the following Eq. (6):

6 $$\frac{{dq}_{t}}{dt}={k}_{2}{\left({q}_{e}-{q}_{t}\right)}^{2}$$

where k_2_ is the PSO adsorption rate constant (mg g^−1^ min^−1^). By separating the variables and then integrating them under the circumstances of q_t_ = 0 at t = 0 and q_t_ = q_e_ at t = t, Eq. (7) can be obtained from Eq. (6). This produces a linear expression that characterizes the adsorption kinetics in a linearised integral form^34^:

7 $$\frac{\text{t}}{{\text{q}}_{\text{t}}}=\frac{1}{{\text{k}}_{2}{\text{q}}_{\text{e}}^{2}}+\frac{1}{{\text{q}}_{\text{e}}}\text{t}$$

**Fig. 22:**
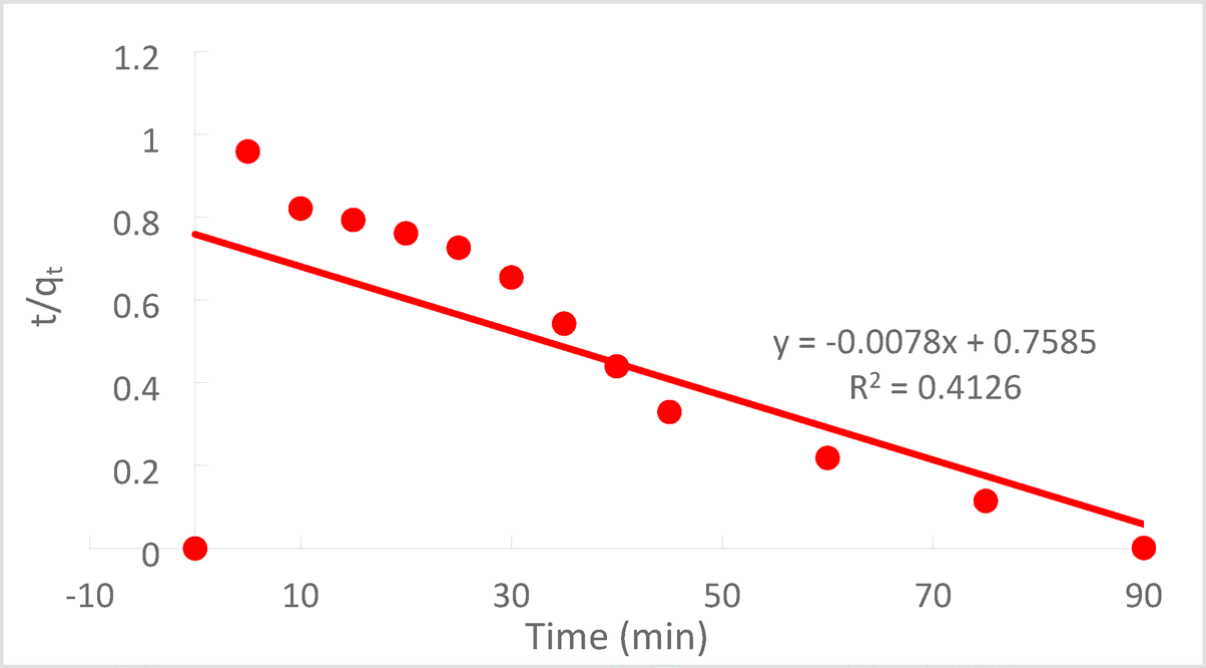
PSF kinetics for the adsorption of Cr^+6^ onto (PFTACs) at(C_o_ = 50 ppm , agitation speed = 300 rpm ,(PFTACs) dose = 0.5 g/250 ml,T = 301 K and t = 90 min,pH = 2).

The ratio of the time over the adsorbed amount of Cr^+6^ (t/q_t_) should be a linear function of time, as shown in Fig. 22, according to the integral form described by Eq. (7). The pseudo-first-order model is more well-behaved than the pseudo-second-order model, according to the correlation coefficients (R_2_) values. This is due to the fact that the pseudo-first-order model’s R_2_ value is marginally greater than the pseudo-second-order model’s^32^.

#### Weber and Morris model (W.M)

Since it establishes the rate of adsorption, it is also referred to as the model of intra-particle diffusion, is important in the study of liquid systems. The kinetics in this model are generally represented by Eq. (8), where the exponent is predicted to be 0.5, and the intercept is a direct function of mass transfer across the boundary layer.

8 $${q}_{t}= {K}_{m}{t}^{0.5}+C$$

where k_m_ is the intra-particle diffusion rate constant (mg/g min^1/2^).

**Fig. 23:**
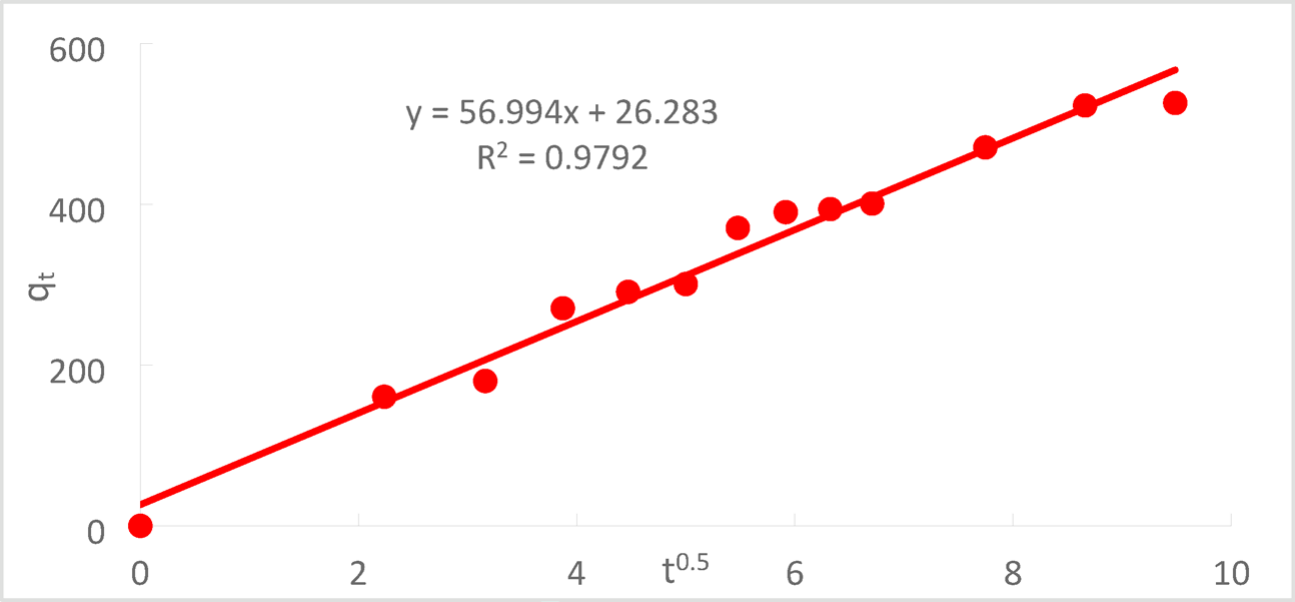
The W.Morris model for Cr^+6^ adsorption onto (PFTACs) at (C_o_ = 50 ppm, agitation speed = 300 rpm,(PFTACs) dose = 0.5 g/250 ml, T = 301 K and t = 90 min, pH = 2).

Figure 23 depicts the plot of q_t_ against t^1/2^, forming a straight line characterized by a slope (k_m_) and an intercept (C). The intercept (C) provides an estimate of the boundary layer thickness, where higher values correspond to a thicker boundary layer^35^.

At C_o_ 50 ppm, contact period of 90 min, agitation rate of 300 rpm, and temperature of 25 °C, the adsorption of Cr^+6^ onto PFTACs was investigated. Table 1 displays the computed outcomes of the kinetics models and associated parameters.

**Table 1:** Kinetic model parameters and the other parameters for adsorption of Cr^+6^ onto (PFTACs) at an initial concentration of Cr^+6^ = 50 (ppm).

Kinetic models	Parameters	Initial concentration (50ppm)
Pseudo first order equation	q_e_ (EXP.)(mg/g)	1362.1
	q_e_ (calc.)	526
	R^2^	0.9931
	10^3^k_1_(min^−1^)	60.5
Pseudo-second-order equation	qe (EXP.)(mg/g)	128.21
	q_e_ (calc.)	526
	R^2^	0.4126
	10^3^k_2_ (g/mg.min)	$8.02\times {10}^{-2}$
Weber and Morris model	C	26.283
	R^2^	0.9792
	K_m_(mg g^−1^ min^−1^)	56.994

### Thermodynamic parameters

Important thermodynamic parameters, such as the standard Gibbs free energy (ΔG°),standard enthalpy change (ΔH°), and standard entropy change (ΔS°), were assessed using the adsorption equilibrium data acquired at various temperatures. Equation (8) was used to determine the standard Gibbs free energy of the Cr^+6^ adsorption process^36^.

9 $$\Delta {G}^{0}=-RTln{K}_{e}$$

The adsorption equilibrium constant (Ke) can be determined for any temperature using Eq. (10).

10 $${K}_{e}=\frac{{q}_{e}}{{C}_{e}}$$

where T is the absolute temperature in Kelvin, C_e_ (mg/L) is the equilibrium concentration of Cr^+6^ in the solution, R is the gas constant (8.314 J/molK), and q_e_ (mg/g) is the quantity of Cr^+6^ adsorbed from the solution at equilibrium.

11 $$\text{Ln }{\text{K}}_{\text{e}}= -\left(\frac{{\Delta \text{H}}^{0}}{\text{RT}}\right)+\left(\frac{{\Delta \text{S}}^{0}}{\text{R}}\right)$$

**Fig. 24:**
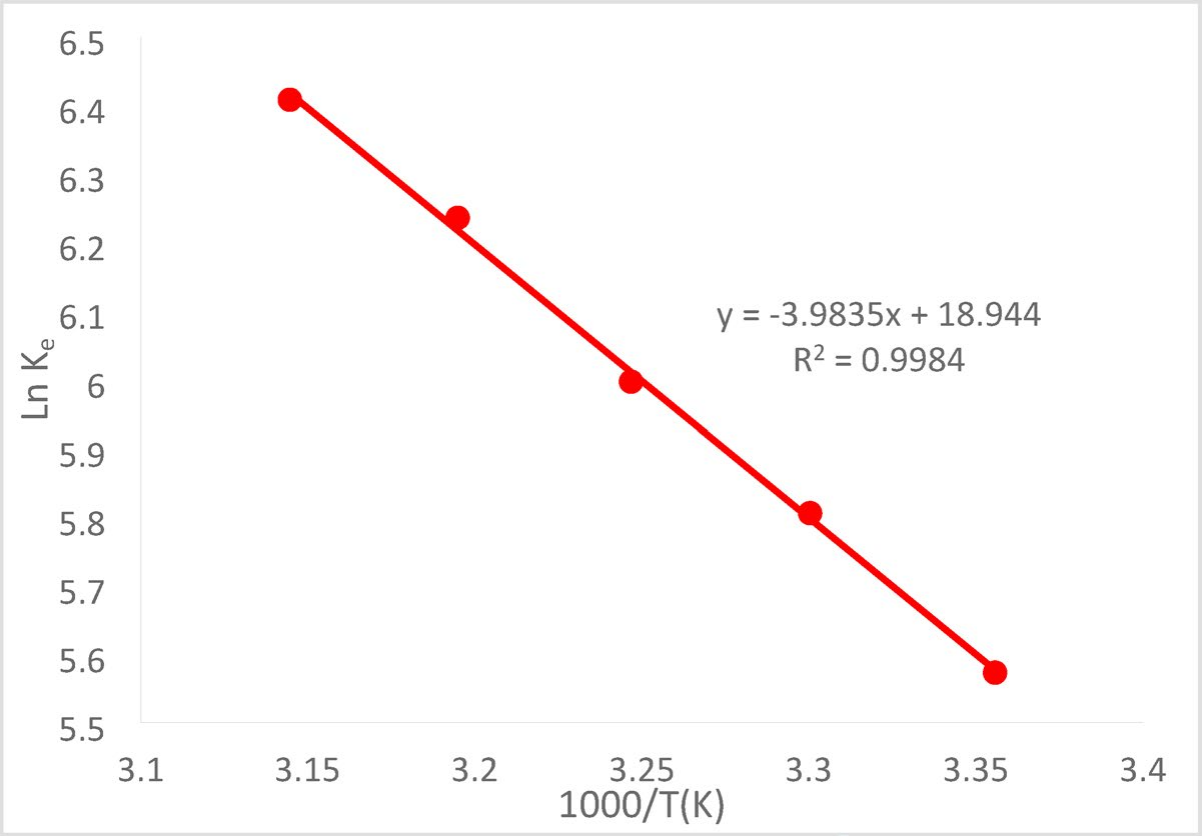
Van̕ t Hoff̓ s plot of the natural logarithm of the adsorption equilibrium constant at C_o_ = 50 ppm, pH = 2, agitation speed = 300 rpm,(PFTACs) dose = 0.5 g /250 ml, t = 90 min at different temperature.

Figure 24 shows the Van’t Hoff plot of 1/T vs. ln(k_eq_) for Cr^+6^, from which ΔS° and ΔH° were derived, respectively, and are shown in Eq. (11).

The values of ΔG°, ΔH°, and ΔS°, which show the energetic characteristics of the divalent Cr^+6^ exchange, are shown in Table 2. While the positive ΔS° value shows a good interaction between the adsorbent and adsorbate, the positive ΔH° value indicates a high energy requirement for the process. A negative ΔG° value, however, suggests that the adsorption process is both possible and spontaneous^26–37^.

**Table 2:** Thermodynamic characterization of Cr^+6^ adsorption at C_o_ = 50 ppm, removed by (PFTACs).

T(K)	298	303	308	313	318
1000/T(K^−1^)	3.355705	3.30033	3.246753	3.194888	3.144654
q_e_	511.0388	549.308	522.26	587.25	554.318
C_e_	1.9412	2.0192	1.539	1.04	0.9132
K_e_	263.26	272.04	339. 4	564.67	607.01
lnK_e_	5.58	5.81	5.997	6.24	6.41
ΔG°(kj/mol)	− 13,807.9	− 14,626	− 15,356.7	− 16,228.4	− 16,943.3
∆H˚(Kj/mol)	33.12				
ΔS°(Kj/mol.K)	157.5				

### Isothermal models

An essential component of determining an adsorbent’s maximal adsorption capacity is the analysis of equilibrium data. In order to assess this capacity, this analysis is necessary. Additionally, since the equation might be used for design purposes, it is crucial to create one that appropriately represents the experimental results. The most often used models for depicting adsorption equilibrium in an adsorption system are the Freundlich and Langmuir equations.

#### Langmuir adsorption isotherm (LAI)

For the solute Cr^+6^, homogeneous surface adsorption was assumed, meaning that monolayer adsorption takes place without any interaction between the adsorbed species. According to the LA hypothesis, the adsorption is monolayer, and the adsorbent’s active sites are uniformly distributed. Chemical adsorption is better explained by this isotherm (9). Equation (13) is the mathematical representation of the Langmuir equation^38,39^:

12 $$\frac{{\text{C}}_{\text{e}}}{{\text{q}}_{\text{e}}}=\frac{1}{{\text{q}}_{\text{max}}\times \text{b}}+\frac{{\text{C}}_{\text{e}}}{{\text{q}}_{\text{max}}}$$

**Fig. 25:**
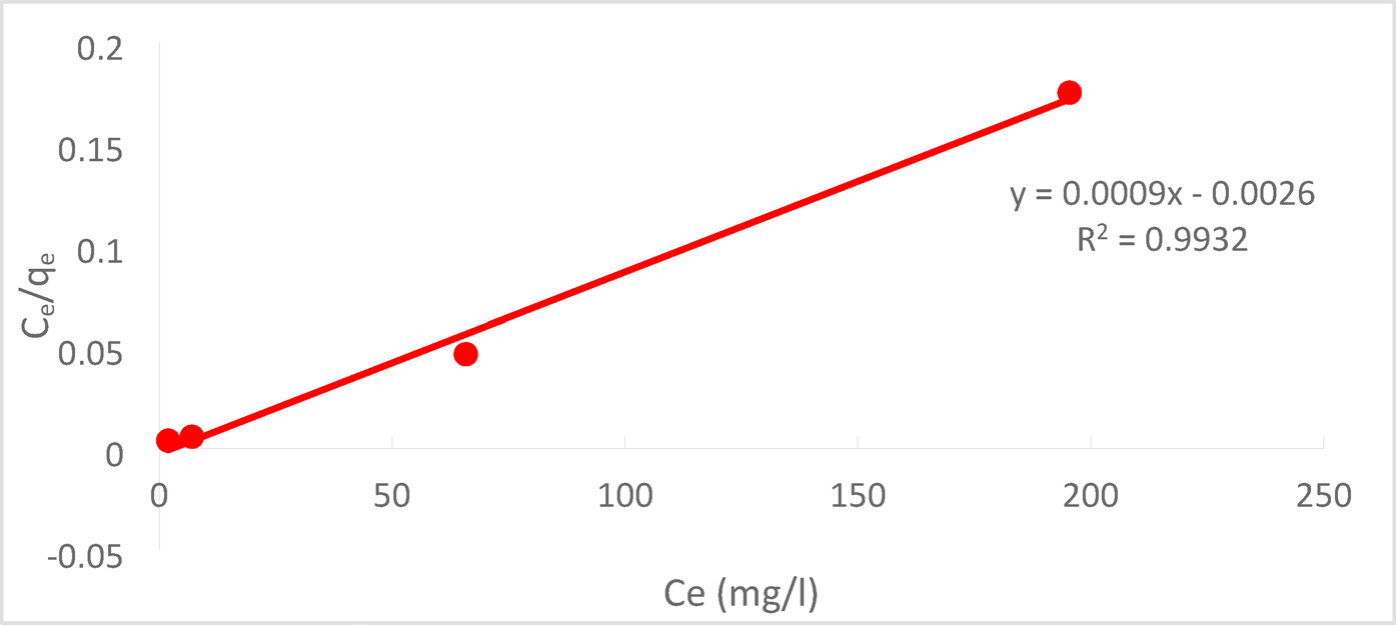
LAI for Cr^+6^ adsorption for the solution of C_o_ = 50 ppm at pH = 2, agitation speed = 300 rpm,(PFTACs) dose 0.5 g /250 ml, t = 90 min T = 25  °C.

The equilibrium concentration of the adsorbate Cr^+6^ on the adsorbent, denoted by the term “q_e_” in the context of adsorption, is given in ppm. “q_max_” is the highest quantity of Cr^+6^ that may adsorb onto the surface of the adsorbent and form a monolayer, whereas “C_e_” denotes the equilibrium concentration of Cr^+6^ in the solution. We call “b” the Langmuir constant. A graphic that illustrates the link between C_e_ and qe is displayed in Fig. 25. The linear relationship between C_e_/q_e_ and C_e_ in this plot indicates that the adsorption of Cr^+6^ follows the Langmuir isotherm model. The reciprocal of q_max_ times b (1/q_max_.b) is represented by the plot’s intercept, and the reciprocal of qmax (1/q_max_) by its slope. A dimensionless constant known as the separation factor or equilibrium parameter, or R_L_, can be used to describe the LI^40^.

13 $${R}_{l}= \frac{1}{1+b\times {C}_{0}}$$

#### Freundlich adsorption isotherm (FAI)

A popular mathematical model for fitting experimental data across a broad range of concentrations is the FAI. Since the active sites of surfaces are heterogeneous, the FI model posits that interactions between adsorbed molecules occur and are not limited to monolayer formation. (12)This isotherm takes into consideration the exponential distribution of active sites and their energies as well as surface heterogeneity. The following is a nonlinear representation of the Freundlich model^41^:

14 $${\text{q}}_{\text{e}}={\text{K}}_{\text{f}} {\left({\text{C}}_{\text{e}}\right)}^{\raisebox{1ex}{$1$}\!\left/ \!\raisebox{-1ex}{$\text{n}$}\right.}$$

**Fig. 26:**
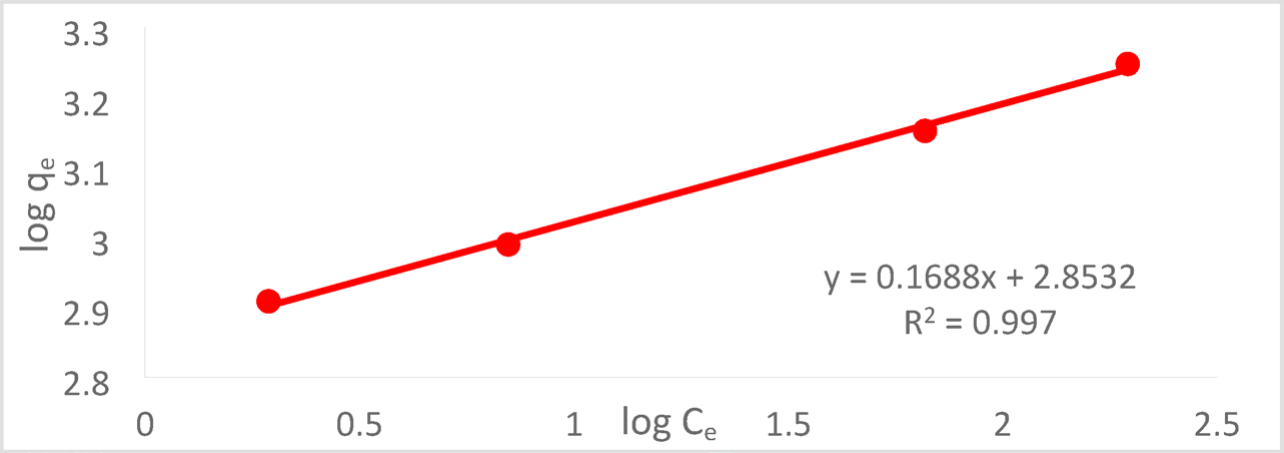
FAI C_o_ = 50 ppm, at a pH of 2, agitation speed = 300 rpm, with a PFTACs dose of 0.5 g per 250 ml, time = 90 min, and T = 25  °C.

The linear form of the Freundlich model is expressed as follows:

15 $$\text{log}{q}_{e}=\text{log}{K}_{f}+\left(\frac{1}{n}\right)\text{log}{C}_{e}$$

where “n” is a constant associated with the sorption intensity that fluctuates according to the heterogeneity of the adsorbent, and “K_f_” is the Freundlich constant signifying the adsorption capacity. As shown in Fig. 26, a linear connection with a slope (1/n) and an intercept (log K_f_) is produced by plotting log qe against log C_e_.

The Freundlich model’s value is closer to 1, suggesting that the chromium adsorption process using the synthesized geopolymer fits this model, even though the correlation (R_2_) values of the two models are near. It is expected that this activity occurs heterogeneously in the material since Freundlich’s model would represent the chromium adsorption process in the synthesized geopolymer^42^.

A comparison of the Freundlich and Langmuir models is shown in Table 3. The maximum monolayer sorption capacity (q_max_) in the Langmuir model dropped as PFTAC dosage increased, while in the Freundlich model.

**Table 3:** LAI constants and FAI constants in the case of Cr^+6^.

	LAI constants			FAI Constants		
Chromium Cr^+6^	q_max_(mg/g)	b (L/mol)	R^2^	1/n	K_f_(mg/g)	R^2^
	1111	− 0.346	0.9932	0.1688	713.18	0.997

The maximal monolayer sorption capacity, or qmax, dropped as PFTAC dosage increased in the Langmuir model but in the Freundlich model.

## Comparison of several adsorbents and pollutant removal

In recent years, the removal of heavy metals and organic pollutants from aqueous environments has become a critical concern due to their toxicity and persistence in ecosystems. Various adsorbents—ranging from natural biomaterials to engineered nanocomposites—have been extensively explored to address this issue. Among these, bio-based activated carbons have gained particular interest due to their low cost, renewability, and high surface functionality. For example, Chitosan-based magnetic nanocomposites have been reported to exhibit remarkable adsorption capacity for heavy metal ions due to their abundant amine and hydroxyl functional groups^43^. Similarly, lignocellulosic-derived carbons have been successfully functionalized to enhance removal efficiency for dyes and metal ions, reinforcing the value of agricultural waste in adsorbent synthesis ^44^.

Moreover, advanced porous materials such as metal–organic frameworks (MOFs), graphene-based composites, and engineered polysaccharides are being widely investigated due to their tailored pore structures and exceptional surface areas, allowing selective and high-capacity adsorption^45^. These advancements reflect the dynamic shift from conventional adsorbents to functionalized, hybrid, and nanostructured materials designed to meet the specific demands of industrial wastewater treatment.

However, challenges persist in balancing adsorption efficiency, regeneration capacity, environmental impact, and production cost. As highlighted by recent reviews, a major research gap lies in scaling up low-cost, bio-derived adsorbents for industrial applications while maintaining performance under variable environmental conditions^46^. For instance, the real-world application of modified biomass and biosorbents is often limited by inconsistent surface chemistry and reduced performance in complex effluent matrices^47^.

Therefore, this study aims to bridge these gaps by developing a sustainable and efficient adsorbent derived from palm fronds an abundant agricultural waste—activated through phosphoric acid treatment. This approach not only utilizes an underexploited biomass but also aims to deliver high adsorption efficiency for hexavalent chromium (Cr^6+^), a known carcinogenic pollutant discharged from various industries. Compared to other bio-adsorbents, palm frond-based activated carbon offers a unique combination of mesoporosity, surface functionality, and low cost, making it a promising candidate for real-world wastewater treatment systems Table 4^48^.

**Table 4:** various adsorbents comparison with PFTACs.

Adsorbent	Adsorption Capacity (%)	Mechanism	Advantages
PFTACs	99.64	Physical adsorption, mesoporous	High adsorption efficiency, low-cost, sustainable, effective in neutral pH
Coconut shell AC	95–98	Physical adsorption, microporous	High adsorption capacity, but more expensive
Biochar	80–90	Surface functionalization	Low-cost, sustainable but less efficient compared to PFTACs
Graphene oxide	99%	Electrostatic, π-π stacking	Very high efficiency, but high production cost
Chitosan-based adsorbents	85–95	Amino group interaction	Biodegradable, but requires careful pH control
ZnO nanoparticles	80–95	Chemical bonding	High efficiency but concerns about stability and recyclability
Clay-based adsorbents	70–85	Ion exchange, surface area	Low cost but needs modification for better efficiency

Looking ahead, the application of such sustainable adsorbents can be extended to treat other industrial pollutants such as textile dyes, pharmaceutical residues, and toxic anions, provided that further functionalization and scale-up studies are conducted. Future research should also focus on multi-contaminant systems, adsorbent regeneration, and life cycle assessment to promote practical andand environmentally responsible use of these materials^49^.

PFTACs exhibit competitive efficiency for Cr^+6^ removal, outperforming many adsorbents in terms of its high adsorption capacity, low cost, and environmentally friendly nature. It presents a sustainable alternative, especially when compared to more expensive materials like graphene oxide or coconut shell activated carbon, making it a promising candidate for large-scale applications in wastewater treatment.

## Conclusion

This study successfully demonstrates the potential of H_3_PO_4_ treated palm frond-derived activated carbon (PFTAC) as an effective and sustainable adsorbent for removing hexavalent chromium (Cr^6+^) from aqueous solutions. The adsorption capacity of PFTACs was found to be 99.64%, achieved under optimal conditions using the batch equilibrium method. Factors such as initial pH, adsorbent dosage, and Cr^6+^ concentration were found to significantly influence the adsorption efficiency, with the highest removal occurring at neutral pH levels.

At pH 2, the removal efficiency was high (99.35%), but decreased to 86.67% as the pH rose to 8. Increasing the initial Cr^+6^ concentration led to a lower removal efficiency due to saturation of adsorption sites. On the other hand, increasing the PFTACs dose improved the removal efficiency, rising from 92.63 to 99.01%. Higher temperatures also enhanced the removal efficiency, with an increase from 97.22 at 25 °C to 99.64% at 45 °C. Kinetic studies showed that both the pseudo-first-order and pseudo-second-order models fit the data, with the former yielding a higher R_2_ value. The Weber and Morris intra-particle diffusion model indicated that mass transfer was a rate-limiting step. Thermodynamic analysis revealed negative ΔG° values, confirming that the process is spontaneous, while positive ΔH° and ΔS° values suggest that the adsorption is endothermic and leads to increased disorder. The adsorption data fit well with the Langmuir isotherm (R_2_ = 0.9932), indicating monolayer adsorption, while the Freundlich isotherm (R_2_ = 0.997) suggested a heterogeneous surface.

The relationship between the weight and surface area (S.A.) of PFTACs further corroborated its high adsorption capacity, demonstrating that a larger surface area corresponds to higher pollutant uptake. FT-IR analysis revealed that the adsorption of Cr^6+^ primarily occurs through physical interactions between the adsorbent and the pollutant, suggesting that the adsorption mechanism is primarily physical rather than chemical.

Both the Freundlich and Langmuir adsorption isotherms were tested, with the Langmuir model providing the best fit, indicating that the adsorption process follows a monolayer adsorption mechanism. This supports the idea that PFTACs exhibit high adsorption capacity and efficiency under controlled conditions.

In conclusion, PFTACs derived from the abundant and renewable resource of palm fronds—offer a sustainable, cost-effective, and efficient solution for the removal of Cr^6+^ from contaminated water, particularly under neutral pH conditions. The results of this study suggest that palm frond-based activated carbon could be a promising material for large-scale wastewater treatment applications, contributing to both environmental sustainability and the reduction of industrial pollutants.

## Data Availability

The datasets used and/or analysed during the current study available from the corresponding author on reasonable request.
